# Constructing Active Sites from Atomic‐Scale Geometrical Engineering in Spinel Oxide Solid Solutions for Efficient and Robust Oxygen Evolution Reaction Electrocatalysts

**DOI:** 10.1002/advs.202101653

**Published:** 2021-07-09

**Authors:** Xin Yue, Xueping Qin, Yangdong Chen, Yang Peng, Caihong Liang, Min Feng, Xinzhuo Qiu, Minhua Shao, Shaoming Huang

**Affiliations:** ^1^ Guangzhou Key Laboratory of Low‐Dimensional Materials and Energy Storage Devices Collaborative Innovation Center of Advanced Energy Materials School of Materials and Energy Guangdong University of Technology Guangzhou 510006 P. R. China; ^2^ Synergy Innovation Institute of GDUT Guangdong University of Technology Heyuan P. R. China; ^3^ Department of Chemical and Biological Engineering The Hong Kong University of Science and Technology Clear Water Bay Kowloon Hong Kong P. R. China; ^4^ HKUST‐Shenzhen Research Institute No. 9 Yuexing 1st RD, South Area, Hi‐tech Park, Nanshan Shenzhen 518057 P. R. China

**Keywords:** cation vacancy, cationic misalignment, lattice strain, oxygen evolution reaction, spinel oxide solid solution

## Abstract

Spinel oxides are considered as promising low‐cost non‐precious metal electrocatalysts for oxygen evolution reaction (OER) due to their desirable catalytic activities and fast kinetics. However, as a result of the structural complexity of spinel oxides, systematic and in‐depth studies on enhancing the OER performance of spinel oxides remain inadequate. In particular, the construction of active sites regarding the large number of unoccupied octahedral interstices has not yet been explored. Herein, more octahedral sites with high OER activities are constructed on the surface of spinel oxides via a cationic misalignment, which is induced by the defects in the spinel oxide solutions, i.e., MoFe_2_O_4_ and CoFe_2_O_4_ nanosheets supported on an iron foam (MCFO NS/IF). With increased active sites and modified electronic structure, the state‐of‐the‐art electrocatalyst exhibits the excellent OER catalytic activity with an onset potential of 1.41 V versus RHE and an overpotential of 290 mV to achieve a current density of 500 mA cm^−2^. Moreover, such an electrocatalyst also demonstrates fast kinetics with the Tafel slope of 38 mV dec^−1^ and superior durability by maintaining the OER activity at 250 mA cm^−2^ for 1000 h.

## Graphical abstract

It is proposed to enhance the catalytic activity of oxygen evolution reaction (OER) by constructing active octahedral sites through cationic misalignment induced by defects in solid solution of MoFe_2_O_4_ and CoFe_2_O_4_ nanosheets (MCFO NS/IF). Based on the increase of active sites and tuning electronic structures, MCFO NS/IF exhibits the excellent catalytic activity of OER.

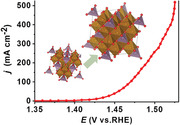

## 1 Introduction

Oxygen evolution reaction (OER) is crucial for various electrochemical energy conversion and storage processes.^[^ ^1^ ^]^ However, OER involves the formation of multiple intermediates with four electron‐proton transfer and the formation of O─O bonds, resulting in sluggish kinetics and large overpotentials to overcome the high energy barrier.^[^ ^2^ ^]^ The high costs, scarce resources, and low stability prevent the utilization of precious‐metal based materials for the alkaline OER application.^[^ ^3^ ^]^ Therefore, it is critical to design and develop cheap and earth‐abundant non‐precious metal electrocatalysts for OER with high activity and stability.^[^ ^4^ ^]^

Transition metal (TM) oxides have been widely regarded as promising and low‐cost OER electrocatalysts due to the variety of their physical and electronic properties.^[^ ^5^ ^]^ Among them, spinel oxides have garnered significant attention owing to their outstanding catalytic performance, thermodynamic stability, excellent conductivity, and environmental friendliness.^[^ ^6^, ^7^ ^]^ The crystal structure of spinel oxide comprises of a cubic close‐packed oxygen anions lattice along with TM cations filling into the octahedral interstices that consist of six oxygen anions and tetrahedral interstices made up of four oxygen anions. In the spinel oxide, half of 32 octahedral sites are occupied by TM (TM_oct_), and one‐eighth of 64 tetrahedral interstices are filled by TM (TM_td_).^[^ ^7^, ^8^ ^]^ As a result, due to their short TM─TM bonds, OER process on spinel oxides involves a multiple‐active‐sites mechanism with faster kinetics than those on other TM oxides, e.g., perovskite.^[^ ^8^, ^9^ ^]^ Generally, the OER activity of spinel oxides mainly originates from TM_oct_ due to the following reasons: (i) Shorter distance between TM_oct_‐TM_oct_ can provide accelerated kinetics for OER through a multiple‐active‐site mechanism with the interaction between redox‐active TM_oct_ and the OER intermediates;^[^ ^6^, ^8^, ^10^, ^11^ ^]^ (ii) It is believed that TM_oct_ is preferentially exposed at the surface of spinel oxides, while the TM_td_ is almost undetectable at the near‐surface.^[^ ^12^ ^]^ Thus, designing and modifying the octahedral geometrical structure is deemed as a feasible strategy to boost the OER performance.^[^ ^6^, ^10^, ^13^ ^]^ Currently, most reports were primarily focused on the doping of heteroatoms and the substitution of ions to enhance the catalytic performance.^[^ ^6^, ^11^ ^]^ Even though there is general improvement in the OER performance of spinel oxides modified via these above strategies, the overall OER performance is still deemed to be less‐than‐satisfactory. To further enhance the intrinsic catalytic activity, constructing more TM_oct_ active sites in spinel oxides by filling the unoccupied octahedral interstices with cations could be a feasible strategy. However, due to the structural complexity of spinel oxides, such a method is often neglected in previous work. Thus, a systematic and in‐depth study to enhance the OER performance of spinel oxides is still lacking as compared to perovskite.^[^ ^11^, ^14^ ^]^

Herein, we design and fabricate a solid solution that is composed of similarly structured MoFe_2_O_4_ (MFO) and CoFe_2_O_4_ (CFO) nanosheets supported on an iron foam (IF) (the sample is denoted as MCFO NS/IF). The formation of Fe cation vacancies in the spinel oxide can be achieved by the oxidation of cations with multiple valence states, such as Mo and V.^[^ ^15^ ^]^ As a result, foreign ions can be anchored onto these vacancy sites, which can easily contribute to the irregularity in the distribution of foreign ions within the lattice.^[^ ^16^ ^]^ Thus, such a process can lead to the random filling of cations into the octahedral interstices due to the anchoring effect of the vacancy sites. Meanwhile, a solid solution is generated by the redistribution of cations due to the tendency of cation substitution in spinel oxide.^[^ ^16^, ^17^, ^18^ ^]^ This process is accompanied by the refilling of the octahedral sites by the cations to increase the amount of TM_oct_ on catalyst surfaces as active sites. In this work, the as‐fabricated MCFO NS/IF electrocatalyst shows an excellent OER activity with an onset potential of 1.41 V versus RHE and an overpotential of 290 mV at the current density of 500 mA cm^−2^. Furthermore, the as‐prepared electrocatalyst also exhibits faster kinetics with the Tafel slope of 38 mV dec^−1^ and superior stability by maintaining the OER activity for 1000 h at a current density of 250 mA cm^−2^.

## 2 Results and Discussion

**Figure 1:**
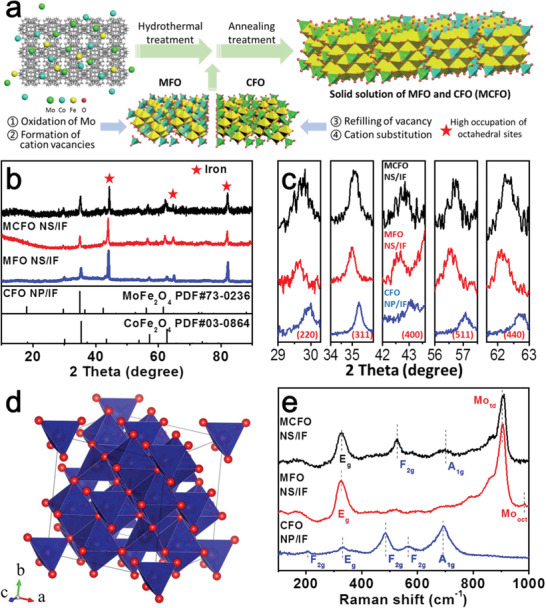
a) Scheme of synthesis route for MCFO NS/IF. b,c) XRD patterns of MCFO NS/IF, MFO NS/IF and CFO NP/IF. d) Crystal structures of cubic spinel oxide. e) Raman spectra of MCFO NS/IF, MFO NS/IF and CFO NP/IF.

The synthesis process of MCFO NS/IF involves a wet‐chemical route and a subsequent annealing treatment as schematically illustrated in **Figure** **1**a. The IF serves as the Fe cation precursor as well as the conductive supporting substrate. Ammonium molybdate tetrahydrate ((NH_4_)_6_Mo_7_O_24_ **·**4H_2_O) and cobalt chloride hexahydrate (CoCl_2_·6H_2_O) were used in the hydrothermal process as the Mo and Co cation precursors, respectively. Sodium dodecyl sulfate (SDS) was added to control the formation of nanosheets.^[^ ^19^ ^]^ As a result, the atomic ratio of Mo to Co in MCFO NS is close to 11:1 (Table S1, Supporting Information). It is worth noting that the spinel oxide phase can be obtained after hydrothermal treatment at above 130 °C for at least 5 h (Figures S1 and S2, Supporting Information). For comparison, MoFe_2_O_4_ nanosheets supported on IF (denoted as MFO NS/IF) and CoFe_2_O_4_ nanoparticles supported on IF (denoted as CFO NP/IF) were prepared as described in the Experimental Section. To obtain a fair comparison with MCFO NS/IF, the amounts of Mo cation in MFO NS/IF and Co cation in CFO NP/IF are equal to those in MCFO NS/IF. Inductively Coupled Plasma‐atomic Emission Spectrometry (ICP) characterization proves that the contents of Mo cations in MFO NS/IF and Co cations in CFO NP/IF are very close to that in the MCFO NS/IF and they are reasonable as comparisons (Table S2, Supporting Information). In this work, the synthesis method of MCFO NS/IF does not introduce foreign organic solvents and rely on high temperature treatment. Meanwhile, this method has the potential for the large‐scale production.

According to the X‐ray diffraction (XRD) pattern of MCFO NS/IF, the peaks observed at 29.7°, 35.2°, 42.9°, 56.9°, and 62.2° can be assigned to (220), (311), (400), (511), and (440) facets of the solid solution of MCFO. All the diffraction peaks of MCFO are loaded between the peaks of the corresponding facets for MFO (PDF#73‐0236) and CFO (PDF#03‐0864), indicating the successful synthesis of a MFO and CFO solid solution (Figure 1b,c). The peaks located at 44.7°, 65.0°, and 82.3° in the XRD patterns of all three samples can be ascribed to the (110), (200), and (211) facets of the Fe substrate (PDF#65‐4899). Both MoFe_2_O_4_ and CoFe_2_O_4_ are comprised of cubic‐spinel structures with the space group of *Fd‐3m(227)* and unit cell parameters of a = b = c = 8.509 and 8.377 Å, respectively (Figure 1d).

The Raman spectrum of CFO NP/IF, as presented in Figure 1e, reveals five Raman active modes (A_1g_+E_g_+3F_2g_), which is in agreement with the Raman active modes of the crystal structure with *Fd‐3m* space group.^[^ ^20^ ^]^ For the Raman spectrum of CFO NP/IF, the Raman peaks located at 485 and 690 cm^−1^ can be assigned to the vibrations of tetrahedral and octahedral sub‐lattice, respectively.^[^ ^21^ ^]^ However, a strong Raman peak located at 907 cm^−1^ can be observed for MFO NS/IF, which can be ascribed to the vibration of O─Mo─O bonds in a MoO_4_ tetrahedral geometry. This result indicates that the dominant occupancy of Mo cation is in the tetrahedral site.^[^ ^22^ ^]^ Interestingly, the Raman spectrum of MCFO NS/IF also reveals the dominant filling of Mo in the tetrahedral interstices, which is similar to that of MFO NS/IF. However, the F_2g_ and A_1g_ modes at 524 and 700 cm^−1^ are blue‐shifted when compared to CFO NS/IF, suggesting the existence of cation substitution in the tetrahedral and octahedral sub‐lattice due to the cationic rearrangement during the solid solution formation.^[^ ^23^ ^]^

**Figure 2:**
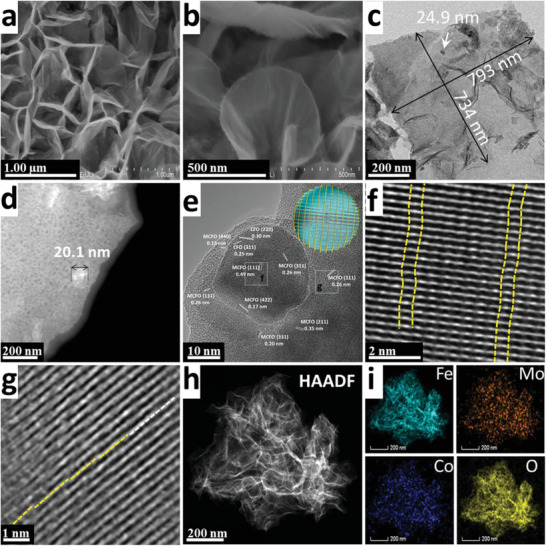
a,b) SEM images of MCFO NS/IF. c) TEM image of MCFO NS. d) HAADF‐STEM image of edge on MCFO NS. e–g) HR‐TEM images of MCFO NS. h,i) HAADF‐STEM and corresponding elemental‐mapping images of MCFO NS.

The morphologies of MCFO NS/IF, MFO NS/IF, and CFO NP/IF are investigated by scanning electron microscope (SEM) and transmission electron microscope (TEM) (**Figure** **2** and Figures S3–S10, Supporting Information). The network‐like porous iron foam acts as a conductive and self‐supporting substrate, which avoids using a binder during the electrode preparation (Figure S3, Supporting Information). The SEM images of MFO NS/IF and CFO NP/IF (Figures S4 and S5, Supporting Information) indicate that MFO NS/IF possesses a nanosheet‐like morphology, while CFO NP/IF shows a nanoparticle‐like morphology with a particle size of 30–150 nm. In contrast, the SEM image of MCFO NP/IF, as presented in Figure 2a, reveals three‐dimensional (3D) flower‐like nanosheet morphology. Such morphology is beneficial to the OER performance since a copious amount of electrolyte can be initially stored in the porous ion‐buffering reservoirs that are composed of NSs. This in turn can provide a rapid supply of reactant and contribute to a short ion‐diffusion pathway.^[^ ^19^ ^]^ The SEM images of the few‐layers and single‐layer MCFO nanosheets are shown in Figure 2b. The Brunauer–Emmett–Teller (BET) characterization is used to investigate the pore structures of MCFO NS and MFO NS. The N_2_ adsorption‐desorption isotherms of MCFO NS and MFO NS can be classified as a typical II isotherm with a hysteresis loop, suggesting the presence of mesoporous structures (Figure S6, Supporting Information).^[^ ^19^ ^]^ The BET specific surface areas of MCFO NS and MFO NS are 10.9 and 8.0 m^2^ g^−1^, respectively. Moreover, it is demonstrated that the 3D flower‐like morphology can be formed via hydrothermal treatment at temperatures as low as 70 °C, and the growth of these 3D flowers occurs with increased temperature. However, when the hydrothermal process is conducted at a lower temperature, i.e., 70 °C, a large number of NPs are formed instead, which gradually disappears as the temperature increases (Figures S7 and S8, Supporting Information). At the same time, the growth and decomposition of a large number of NPs have been observed with rising the time of hydrothermal treatment (Figure S9, Supporting Information). This result clearly indicates that the formation of MCFO NS after the decomposition of CFO NPs, which leads to the redistribution of Co and Fe cations into MFO NSs.

A typical top‐view TEM image of the MCFO NS with a dimension of 734 × 793 nm is shown in Figure 2c. As indicated in Figures S10 and S11 (Supporting Information), the TEM images of MFO NSs and CFO NPs show that the typical MFO NS consists of NPs with a size of about 5 nm, while the average particle size of CFO is larger than 30 nm. Since both MCFO and CFO possess similar crystal structures, they can only be distinguished by the small differences in the lattice distance of the same facets and different particle sizes. According to the TEM image (Figure 2c) and high angle annular dark field‐scanning transmission electron microscope (HAADF‐STEM) image (Figure 2d), MCFO NS is composed of MCFO NPs with an average particle size of about 5 nm, together with a small amount of incompletely decomposed CFO NPs (with size larger than 20 nm) as residue. These observations for MCFO NS are consistent with the average particle size of MFO and CFO. Figure 2e shows the high resolution (HR) TEM image of the edge of MCFO NS. The marked particle with a size of about 27 nm can be identified as the CFO NP. This is due to the matching interplanar distances of 0.30 and 0.15 nm to their corresponding (220) and (311) facets of the CFO, and the 31° angle between these two facets, which is consistent with the result obtained from the selected area electron diffraction (SAED) (Figure S12b, Supporting Information). However, it is inferred that the three crystalline indices of 0.49, 0.26, and 0.17 nm that appeared for CFO NP correspond to (111), (311), and (422) facets of MCFO, based on the slightly larger interplanar distances and included angles (Figure S12c–e, Supporting Information). The inset of Figure 2e shows the schematic illustration of the possible structure of CFO covered by nanosheets that are composed of smaller MCFO NPs, leading to the observation of both crystal lattices. Other crystalline indices on the NS can be confirmed from MCFO, whereby interplanar distance of 0.20, 0.26, 0.35, and 0.15 nm correspond to (331), (311), (211), and (440) facets, respectively (Figure S12f–i, Supporting Information). The corresponding marked square regions in Figure 2e are magnified and shown in Figure 2f,g, respectively. The lattice distortion in the MCFO can be observed clearly in Figure 2f, whereby such a distortion may be attributed to the lattice‐mismatch arising from the misalignment of cations with different radii.^[^ ^16^, ^17^ ^]^ Furthermore, stacking faults in the lattice of MCFO can be observed, implying the existence of lattice strain. Such a lattice strain in MCFO is caused by the formation of cation vacancies that is followed by the proceeding of Mo cation oxidation from the surface to the core of the grains and the subsequent substitution of cations with different radii.^[^ ^16^, ^24^ ^]^ According to the HAADF‐STEM and corresponding elemental mapping images, uniform distributions of Fe, Mo, Co, and O can be observed across MCFO NS (Figure 2h,i), which indicates the successful formation of MCFO.

**Figure 3:**
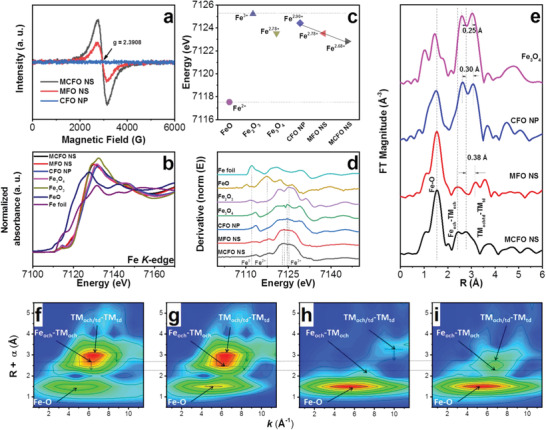
a) The electron paramagnetic resonance (EPR) spectra of MCFO NS, MFO NS and CFO NP. b) Normalized XANES spectra at the Fe K‐edge of MCFO NS, MFO NS, CFO NP, and comparisons. c) Relationship between valence states and absorption energy in the first derivative of XANES spectra at Fe K‐edge of MCFO NS, MFO NS, CFO NP, and reference materials. d) The first derivative of XANES spectra at Fe K‐edge of MCFO NS, MFO NS, CFO NP, and comparisons. e) Resulting Fourier‐transformed (FT) *k^3^* weighted *χ*(*k*)‐function of Fe K‐edge EXAFS spectra for Fe_3_O_4_, CFO NP, MFO NS, and MCFO NS. Wavelet transforms (WT) for the *k^3^* weighted EXAFS contour plots of f) Fe_3_O_4_; g) CFO NP; h) MFO NS; and i) MCFO NS.

The X‐ray photoelectron spectroscopy (XPS) results in Figure S13 (Supporting Information) imply an increased content of Fe_oct_ in the MCFO NS due to the negative shift in the binding energy.^[^ ^25^ ^]^ On the other hand, Co cations almost only occupy the tetrahedral sites in MCFO NS and CFO NP (Figures S13–S16, Supporting Information). Generally, the valence states of Mo cations in MFO are mainly Mo^3+^ and Mo^4+^.^[^ ^26^ ^]^ Therefore, the increase in the average valence state of Mo cation in MCFO NS is mainly caused by the oxidation of Mo_oct_ located at the near‐surface due to their preferential exposures of TM_oct_ to the near‐surface, which indicates that more Mo cations occupy the octahedral sites (Figure S15, Supporting Information).^[^ ^12^ ^]^ Thus, this result indicates that there is an increase in the occupancy of TM_oct_ in the MCFO. As known, the electron paramagnetic resonance (EPR) spectroscopy is an important tool for probing the unpaired electron states in materials to verify the existence of the defects.^[^ ^27^ ^]^ The EPR spectra of MCFO NS, MFO NS and CFO NP are shown in **Figure** **3**a. The signal of MCFO NS is obviously more intensified and broadened than that of MFO NS at *g *= 2.3908, indicating the existence of more Fe vacancies.^[^ ^27^ ^]^ However, almost no signal is detected on the EPR spectrum of CFO NP, implying that there are almost no Fe vacancies in CFO NP. In other words, it confirms that the formation of Fe vacancies in the MCFO NS and MFO NS can be achieved by the oxidation of Mo cations.

Furthermore, to gain a deeper insight into the valence states and coordination environments of various spinel oxides, X‐ray absorption fine structure (XAFS) measurements were conducted. X‐ray absorption near edge structure (XANES) spectra were recorded at the Fe K‐edge of MCFO NS, MFO NS, CFO NP, and other compounds (Figure 3b).^[^ ^28^, ^29^ ^]^ The XANES spectra of FeO and Fe_2_O_3_ show that white line intensities are located at 7127.8 and 7132.5 eV, corresponding to Fe^2+^ and Fe^3+^, respectively. Among the various spinel oxides, CFO NP exhibits a white line intensity at 7132.4 eV, demonstrating that the valence state of Fe in CFO NP is the highest, which is close to Fe^3+^. In contrast, MCFO NS and MFO NS show white line intensities at 7129.9 eV, which implies that both of these samples possess the lowest Fe valence state among the various spinel oxides. The valence state of Fe can be analyzed from the absorption energy positions of the highest peak of the first derivative of XANES spectra at Fe K‐edge (Figure 3c,d).^[^ ^6^, ^30^ ^]^ As shown in Figure 3c, the average valence state of Fe in the MCFO NS is 2.68+, which is lower than those in MFO (2.78+) and CFO (2.90+). Such a result indicates that the cation substitution and misalignment can induce a change in the coordination environment of the Fe cation.^[^ ^6^ ^]^

As observed in the Fourier transform (FT) Fe K‐edge extended X‐ray absorption fine structure (EXAFS) spectra (Figure 3e), the first peak at around 1.5 Å represents the Fe─O bonds.^[^ ^8^, ^31^ ^]^ The peak at ≈2.5 Å corresponds to the bonds between the nearest octahedral cations, i.e., Fe_oct_‐TM_oct_, and the peak at ≈3.0 Å is attributed to the bonding of the nearest tetrahedral cations, i.e., Fe_td_‐TM_td_, or the bonding between the tetrahedral cation with the nearest octahedral cations (Fe_td_‐TM_oct_ or Fe_oct_‐TM_td_).^[^ ^8^, ^28^ ^]^ There is a sharp decline in the amplitudes of the characteristic peaks for TM_oct_ and TM_td_ in the EXAFS spectrum of MFO NS, which implies a lower metal coordination number. Thus, this result illustrates the existence of cation vacancies in MFO NS.^[^ ^31^ ^]^ Although based on the XPS results (Figure S15, Supporting Information), the increasing valence state of Mo indicates the formation of more Fe cation vacancies, there is an increase in the ratio of the characteristic peak intensities of TM_oct_ and TM_td_ to the peak intensity of Fe─O bond in the EXAFS spectrum of MCFO NS as compared to that of MFO NS. This result indicates that the Fe cation vacancies are refilled in MCFO NS. The positions of the characteristic peak of Fe_oct_‐TM_oct_ for various spinel oxides are close to 2.5 Å, i.e., Fe_3_O_4_ (2.59 Å), CFO NP (2.62 Å), MFO NS (2.38 Å), and MCFO NS (2.42 Å). However, the peak position of TM_td_ in the EXAFS spectrum of MCFO NS exhibits a left‐shift to 2.78 Å, which is 0.38, 0.30, and 0.25 Å smaller than those of MFO NS, CFO NP, and Fe_3_O_4_, respectively. The unaltered distance of Fe_oct_‐TM_oct_ and the reduced bond length of Tm_td_‐TM_td/oct_ demonstrate the presence of incorrect filling of cations into unoccupied interstices (Figure S17, Supporting Information). Thus, based on the XPS results, TM cations are mainly filled into the unoccupied octahedral sites.

Wavelet transforms (WT) of the *k^3^* weighted EXAFS at Fe K‐edge of various spinel oxides are shown in Figure 3f–i. In general, the coordination features of heavy elements are located in regions with higher *k* values.^[^ ^32^ ^]^ Coordination features of TM_oct_‐TM_oct_ and TM_td_‐TM_td/oct_ in Fe_3_O_4_ and CFO NP are correlated with a *k* value of about 6 Å^−1^, which indicates a close approximation in the *k* values between Fe and Co. Simultaneously, the maximum intensity is located at about 9.5 Å^−1^ in the WT‐EXAFS contour plot of MFO NS, which is attributed to the Fe─Mo bond. According to the Raman and XPS results, it can be confirmed to be the Fe_oct_─Mo_td_ bond due to the dominant occupancy of Mo cations in the tetrahedral interstices. However, the maximum intensity of the characteristic Fe_oct_─TM_oct_ bond shifts to 7 Å^−1^, which implies the filling of the octahedral interstices by Mo cations. According to the WT EXAFS contour plot of MCFO NS, the maximum intensities located at about 2.3 and 2.7 Å can be both correlated with a *k* value of ≈7 Å^−1^. This result proves the reduction of the TM_td/oct_─TM_td_ bond, and also indicates that the cationic misalignment in the lattice is dominated by the incorrect filling of unoccupied octahedral interstices in MCFO by Fe cations.

**Figure 4:**
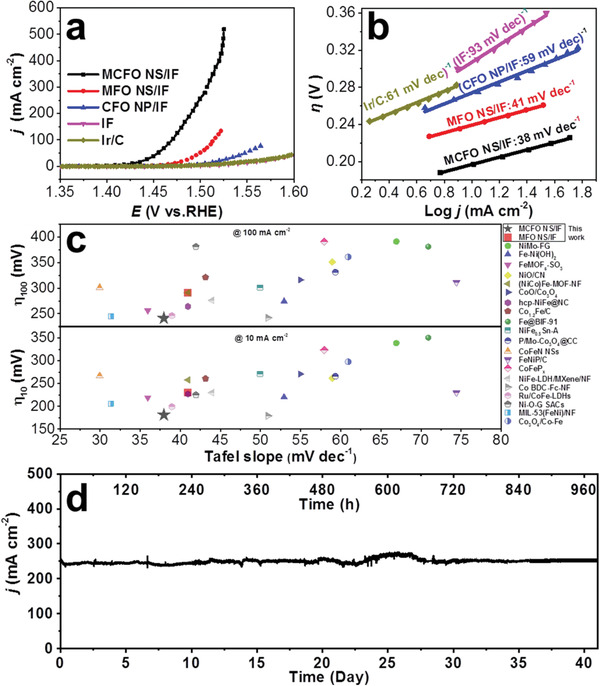
a) Linear sweep voltammogram (LSV) curves of OER on MCFO NS/IF, MFO NS/IF, CFO NP/IF, Ir/C, and IF electrocatalysts. LSV curves were performed in 1.0 m KOH at 25 °C with a scan rate of 0.5 mV s^−1^. b) Tafel plots of MCFO NS/IF, MFO NS/IF, CFO NP/IF, Ir/C, and IF electrocatalysts. c) Comparison of OER activities in Tafel slopes and overpotentials on MCFO NS/IF and reported state‐of‐the‐art electrocatalysts. d) The chronoamperometric plot of OER on MCFO NS/IF at 1.49 V versus RHE in 1.0 m KOH for 1000 h (25 °C).

To investigate the enhancement in the catalytic activity that arises from the geometrical modifications in MCFO NS/IF electrocatalyst, OER polarization curves were measured. For comparisons, LSV curves of MFO NS/IF, CFO NP/IF, Ir/C and IF electrocatalysts were collected and shown in **Figure** **4**a. It is worth noting that all polarization curves were *iR*‐corrected. It can be observed that MCFO NS/IF exhibits an onset potential of 1.41 V versus RHE, which is 50 and 70 mV lower than those of MFO NS/IF and CFO NP/IF, respectively. MCFO NS/IF reaches a current density of 100 mA cm^−2^ at only 1.47 V versus RHE, which is about 50 mV lower than that of MFO NS/IF. Furthermore, MCFO NS/IF only requires an overpotential of 290 mV to achieve a current density of 500 mA cm^−2^. It should be noted that the IF electrocatalyst shows a negligible catalytic activity, demonstrating that the IF substrate should not contribute to the overall OER performance of samples. The catalytic current density of OER on Ir/C is only 44.5 mA cm^−2^ even at 1.60 V versus RHE, implying a negligible activity compared with that of MCFO NS/IF. Interestingly, the MCFO NS/IF heat‐treated at 400 °C exhibits the optimal OER catalytic activity (Figure S18, Supporting Information). The OER current density of MCFO NS/IF is even significantly higher than the sum of current densities of MFO NS/IF and CFO NP/IF at the same overpotential (Figure S19, Supporting Information). This result confirms that constructing more active TM_oct_ sites by geometrical engineering of MCFO can lead to the significant enhancement of OER performances. As a result of the multiple‐active‐sites OER mechanism for spinel oxide electrocatalysts, MCFO NS/IF, MFO NS/IF, and CFO NP/IF are all able to exhibit faster kinetics with lower Tafel slopes (Figure 4b).^[^ ^8^ ^]^ Among them, MCFO NS/IF possesses the lowest Tafel slope of only 38 mV dec^−1^, which indicates the accelerated kinetics during OER. The Tafel slope of MCFO NS/IF is even lower than that of the Ir/C. Furthermore, MCFO NS/IF possesses a larger electrochemical active surface area (ECSA) and minimum reaction resistance as compared to MFO NS/IF and CFO NP/IF, indicating that there are more exposed active sites as well as faster charge transfer kinetics in the spinel oxide solid solution and thereby resulting in better catalytic performance (Figures S20–23, Supporting Information). Consequently, the as‐prepared MCFO NS/IF exhibits a similar level of OER performance in terms of kinetics and overpotentials compared to those recently reported state‐of‐the‐art OER electrocatalysts (Figure 4c, Table S3, Supporting Information). The durability of MCFO NS/IF was also examined, where the OER current density of MCFO NS/IF could be maintained at 250 mA cm^−2^ for 1000 h (about 42 days) (Figure 4d). The fluctuation of the chronoamperometric plot is based on the productions of O_2_ bubbles and the replacement of electrolyte for every 3–5 days. The XRD, XPS and SEM characterizations confirm the robust structural stability of MCFO NS/IF in OER process (Figures S24–26, Supporting Information). Such an unprecedented result may be due to the thermodynamic stability of the spinel oxides, which suggests that the structural stability of the solid solution has not been destroyed even after the geometrical modification.

**Figure 5:**
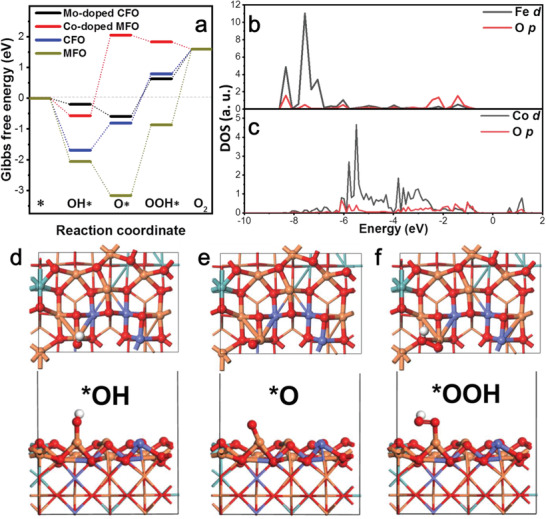
a) Gibbs free energy diagram of OER on various electrocatalyst surfaces, including MFO, CFO, and other two modified model catalysts (Co‐doped MFO and Mo‐doped CFO). b) The curve of projected density of state (PDOS) of Fe *d* orbital and O *p* orbital for *OH on Mo‐doped CFO. c) The curve of projected density of state (PDOS) of Co *d* orbital and O *p* orbital for *OH on CFO. The optimized structures of key intermediates during OER on Mo‐doped CFO: d) *OH; e) *O; f) *OOH. Color code: orange, Fe; blue, Co; teal, Mo; red, O; white, H.

To identify the origin of the performance enhancement in the solid solution, density functional theory (DFT) simulations were performed to investigate the true active sites and the OER mechanisms (**Figure** **5**, Figures S27, S28, S33, and S34, Supporting Information). Four types of computational models were constructed including MFO, CFO, Co‐doped MFO, and Mo‐doped CFO. It has been found the limited promotion of OER activity on the interface of heterostructures between MCFO and CFO (Figures S26–S32, Supporting Information). Thus, the model of Mo‐doped CFO has been constructed to describe the interface of heterostructures between MCFO and CFO. The model of Co‐doped MFO is used to describe not only the solid solution of MCFO, but also the interface of heterostructures between MCFO and CFO. During OER on catalyst surfaces in the alkaline medium, the key reaction intermediates consist of *OH, *O, and *OOH (* denotes adsorbed state). As shown in Figure 5a, the Gibbs free energy diagram is constructed based on the optimized structures and energies of the key intermediates adsorbed on catalyst surfaces. By comparing the energy barriers of the elementary steps during the OER process, it is concluded that the Mo‐doped CFO is the most promising catalyst as it possesses the lowest energy barrier of 1.22 eV. On the other hand, MFO and CFO both show higher energy barriers of 2.46 and 1.60 eV, respectively. It should be noted that the potential‐determining step (PDS) is *O‐to‐*OOH, as it is the same on the surfaces of the three abovementioned catalysts, i.e., MFO, CFO, and Mo‐doped CFO. Another modified model, Co‐doped MFO, was also considered to investigate the possibility of the formation of active sites in the solid solution. The calculation result indicates that there is a shift in the PDS from *OH‐ to *O with a high energy barrier of 2.62 eV due to the significantly weaker binding to *O. The projected density of state (PDOS) was also calculated to provide insights into the difference in the *OH binding strength on Mo‐doped CFO and pure CFO, which are the two model catalysts with lower energy barriers. The PDOS of the *d* orbital of the active sites (Fe in Mo‐doped CFO and Co in CFO) and O *p* orbital of *OH are plotted in Figure 5b,c, respectively. The most downshifted *d*‐band center illustrates a weaker binding between the *OH intermediate and Mo‐doped CFO as compared to that for pure CFO (Figure 5b,c, Table S4, Supporting Information). The active site was determined and the optimized structures of the intermediates on the surface of the most promising catalyst, i.e., Mo‐doped CFO, are shown in Figure 5d,e,f, whereby the exposed Fe_oct_ is the active site (other models on MFO and CFO can be found in Figures S33 and S34 and Table S5 (Supporting Information). Such an active site demonstrates that the exposed Fe_oct_ can contribute to a higher OER activity. Based on the above results, it is illustrated that the cationic misalignment and substitution in MCFO and the interface of heterostructures between MCFO and CFO contribute to the modification of the electronic structures. The modified electronic structure of catalysts can optimize the bindings to key reaction intermediates, thus facilitating the OER process and ultimately leading to the improved catalytic performance as observed in this work.

## 3 Conclusion

In summary, a solid solution comprising of MoFe_2_O_4_ and CoFe_2_O_4_ nanosheets supported on iron foam (MCFO NS/IF) has been successfully prepared via a wet‐chemical route and a subsequent annealing treatment. The cation vacancies that are induced by the oxidation of Mo cations can offer an opportunity for the cations to fill into the unoccupied octahedral interstices. Simultaneously, the substitution and distribution of cation can occur during the generation of solid solution, which is accompanied by the refilling of the octahedral sites by these cations to increase the number of TM_oct_ active sites on catalyst surfaces. According to the HR‐TEM characterization, lattice‐strain along with the cationic misalignment is induced by defects in the materials, such as cation vacancies and substitutions. XAFS measurements reveal the changes in the bond length and coordination environment due to the filling of unoccupied octahedral sites, which confirms the increased number of TM_oct_ active sites. As a result, the as‐prepared material possesses more TM_oct_ active sites that are constructed by the atomic‐scale geometrical engineering. The as‐prepared MCFO NS/IF exhibits excellent catalytic activity (an overpotential of only 290 mV to achieve a current density of 500 mA cm^−2^), fast kinetics (Tafel slope of 38 mV dec^−1^), and high durability (maintaining at 250 mA cm^−2^ for 1000 h). According to the DFT calculations, cationic misalignment and substitution can modify the electronic structures of catalysts, and therefore contribute to the optimized bindings to key reaction intermediates during OER, ultimately leading to the improved catalytic performance. Thus, this study provides a new opportunity for the design and construction of active sites in TM oxides to realize high efficient non‐noble metal OER electrocatalysts.

## 4 Experimental Section

### Synthesis

#### Chemicals

Ammonium molybdate ((NH_4_)_6_Mo_7_O_24_ **·**4H_2_O, AR), cobalt chloride (CoCl_2_ **·**6H_2_O, AR), sodium dodecyl sulfate (SDS, C_12_H_25_O_4_NaS, AR), and potassium hydroxide (KOH, AR) were purchased from Shanghai Titan Scientific Co., Ltd and Shanghai Macklin Biochemical Co., Ltd. Iron foams were purchased from Kunshan Jiayisheng Electronics Co., Ltd. Hydrochloric acid (HCl, AR) was purchased from Chengdu Cologne Chemical Co., Ltd. The deionized water was home‐made.

#### Synthesis of the Solid Solution Comprising of MoFe_2_O_4_ and CoFe_2_O_4_ Nanosheets Supported on Iron Foam (MCFO NS/IF)

Firstly, IF (2.0 × 2.0 cm) was ultrasonic treated 1 m HCl solution for 10 min and then washed in DI water until pH value = 7. The process was repeated three times. Secondly, (NH_4_)_6_Mo_7_O_24_ **·**4H_2_O (0.010 mmol), CoCl_2_ **·**6H_2_O (0.006 mmol), and SDS (0.300 mmol) were ultrasonic treated and dissolved in 60 mL DI water. Then the mixture was completely transferred into a 100 mL Teflon‐lined autoclave, and the pretreated IF (2.0 × 2.0 cm) was put into the mixture. Thirdly, the autoclave was heat‐treated in an electric oven at 150 °C and for 6 h. After cooling down to room temperature, the resulting mixture was washed in DI water three times and dried at 60 °C for 12 h. Finally, the resulting sample was heat‐treated in N_2_ atmosphere at 400 °C for 2 h.

#### Synthesis of MoFe_2_O_4_ Nanosheets Supported on Iron Foam (MFO NS/IF)

(NH_4_)_6_Mo_7_O_24_ **·**4H_2_O (0.010 mmol) and SDS (0.300 mmol) were used. Other synthesizing processes were as same as that of MCFO NS/IF.

#### Synthesis of CoFe_2_O_4_ Nanoparticles Supported on Iron Foam (CFO NP/IF)

CoCl_2_ **·**6H_2_O (0.006 mmol) and SDS (0.300 mmol) were used. Other synthesizing processes are as same as that of MCFO NS/IF.

#### Characterizations

X‐ray diffraction (XRD) measurements were carried on a D‐MAX 2200 VPC diffractometer (Rigaku Co., Japan) with Cu Ka radiation (*λ* = 1.54056 Å) as the radiation source at 40 kV and 40 mA with a scanning rate of 10° min^−1^. Scanning Electron Microscopy (SEM) measurements were taken on a HITACHI SU8010 scanning electron microscope. Transmission Electron Microscopy (TEM) was performed on a Tecnai G2 F20 transmission electron microscope at 200 kV. Raman analysis was performed on a micro‐Raman spectrometer (Renishaw inVia, U.K.). The X‐ray photoelectron spectroscopy (XPS) measurements were carried out on a XPS apparatus (ESCALAB 250, Thermo‐VG Scientific Ltd.).

X‐ray absorption measurements were carried on at Fe K‐edge of various composites at room temperature in the transmission mode with silicon drift fluorescence detector at beam line TLS07A1 of National Synchrotron Radiation Research Center (NSRRC) operated with a Si (111) double crystal monochromator. The synchrotron was detected at 1.5 GeV and 250 mA. MCFO NS, MFO NS and CFO NP were separated from IF by ultrasonic treating in alcohol for over 6 h before XAFS measurement. The photon energy was calibrated with the first inflection point of Fe K‐edge in Fe metal foil. All acquired data were processed and analyzed by Athena and Artemis software.^[^ ^33^ ^]^

#### Electrochemical Measurements

Electrochemical measurements of OER were performed in a typical three‐electrode system. The reversible hydrogen electrode (RHE) was used as the reference electrode and a graphite rod was used as the counter electrode. MCFO NS/IF, MFO NS/IF, CFO NP/IF and IF with a size of 1.0 × 1.0 cm were used as working electrodes, respectively. Electrochemical measurements were performed on an Autolab PGSTAT204 electrochemical potentiostat. Commercial Ir/C (20%, Premetek Co.) electrocatalysts have been used as comparison to study the OER performance on precious metal based catalysts. Ir/C catalysts were added to 1.5 mL ethanol and 0.5 mL Nafion (0.5 wt%, DuPont, USA) and then sonicated for 1 h to form a well‐dispersed electrocatalyst ink. A drop of the electrocatalyst ink was transferred to the surface of the glassy carbon electrode (5 mm in diameter) and dried using an infrared lamp to form an electrocatalyst thin film electrode. The loading of Ir on electrode was 0.102 mg cm^−2^. Polarization curves were tested in 1 m KOH solution at 25 °C with a scan rate of 0.5 mV s^−1^. The stability tests were using chronopotentiometric measurements at 1.49 V versus RHE for 1000 h. All the polarization curves were iR‐corrected.

#### DFT Calculations

The first‐principle calculations were performed within the density functional theory (DFT) formalism as implemented in the VASP code.^[^ ^34^ ^]^ The generalized gradient approximation with the Perdew–Burke–Ernzerhof (PBE)^[^ ^35^ ^]^ realization was used. The interaction between the core and valence electrons was described by the projector‐augmented wave method.^[^ ^36^ ^]^ The kinetic energy cutoff was set to 400 eV. Since Fe, Mo and Co are transition metals and the Coulomb potential is significant, the Hubbard U values of Fe, Mo and Co were set 4, 2, and 4 eV, respectively. The atomic positions were optimized using the conjugate gradient method with total energy and maximum force convergence criteria set as 10^−4^ eV per atom and −0.05 eV Å^−1^, respectively. A (3 × 3 × 1) k‐points grid according to Monkhorst–Pack scheme was used to sample the Brillouin Zone.

## Conflict of Interest

The authors declare no conflict of interest.

## Supporting information

Supporting Information

## Data Availability

Research data are not shared.
