# Supplementary material for: Constructing Active Sites from Atomic‐Scale Geometrical Engineering in Spinel Oxide Solid Solutions for Efficient and Robust Oxygen Evolution Reaction Electrocatalysts
Source: Adv Sci (Weinh). 2021 Jul 9;8(17):2101653. doi: 10.1002/advs.202101653 (PMC8425945; doi:10.1002/advs.202101653)
Supplement: Supplementary file 1 — Supporting Information [file ADVS-8-2101653-s001.pdf]

WILEY-VCH

© 2021 Wiley-VCH GmbH

ADVANCED SCIENCE
Open Access

Supporting Information

for *Adv. Sci.*, DOI: 10.1002/advs.202101653

Constructing Active Sites from Atomic-Scale Geometrical Engineering in Spinel Oxide Solid Solutions for Efficient and Robust Oxygen Evolution Reaction Electrocatalysts

Xin Yue, Xueping Qin, Yangdong Chen, Yang Peng, Caihong Liang, Min Feng, Xinzhuo Qiu, Minhua Shao* and Shaoming Huang**

Supporting Information

Constructing Active Sites from Atomic-Scale Geometrical Engineering in Spinel Oxide Solid Solutions for Efficient and Robust Oxygen Evolution Reaction Electrocatalysts

Xin Yue, Xueping Qin, Yangdong Chen, Yang Peng, Caihong Liang, Min Feng, Xinzhuo Qiu, Minhua Shao* and Shaoming Huang**

XRD patterns of samples only by hydrothermal treated from 100 to 150 °C for 6 h are shown in **Figure S1**. No peaks are appearing XRD patterns of samples treated from 100-120 °C. Diffraction peaks loaded at 35.1 and 62.2° appear in XRD patterns of samples treated at 130-150 °C, indicating the successfully generating for the solid solution of MFO and CFO. It means that phase of spinel oxides can be produced above 130 °C at hydrothermal process. However, signals of XRD patterns are relatively weak, representing lower crystalline degrees.

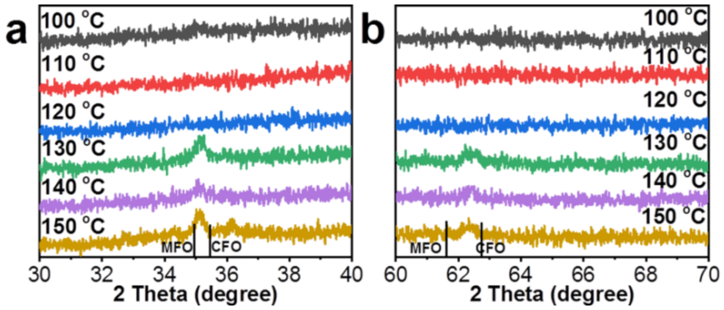

Figure S1 (a, b) XRD patterns of samples only by hydrothermal treated from 100 to 150 °C for 6 h.

XRD patterns of samples only by hydrothermal treated at 150 °C for different times from 1 to 5 h are shown in the **Figure S2**. Diffraction peaks loaded at 35.1 and 62.2° only appear in XRD patterns of samples treated for 5 h.

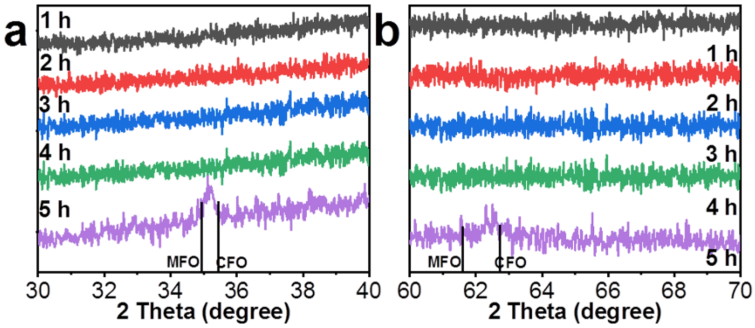

Figure S2 (a, b) XRD patterns of samples only by hydrothermal treated at 150 °C for different times from 1 to 5 h.

Figure S3 shows SEM images of iron foam. Porous IF was used as the precursor of Fe cations and conductive supporting materials. Meanwhile, porous structure is beneficial for the adsorption of adsorbate on contactable active sites.

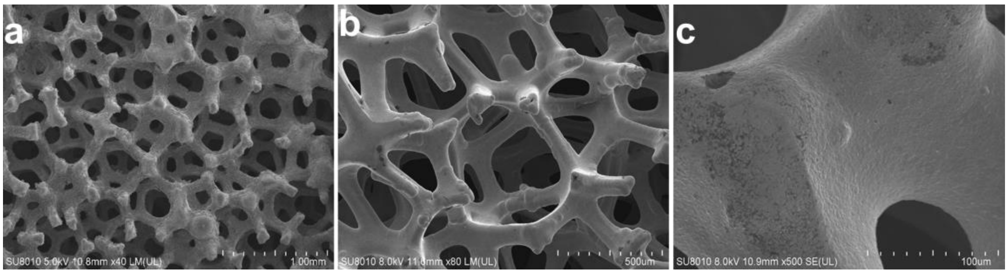

Figure S3 SEM images of iron foam.

SEM images of MFO NS/IF are shown in the **Figure S4**. MFO NS/IF exhibits morphology of nanosheets.

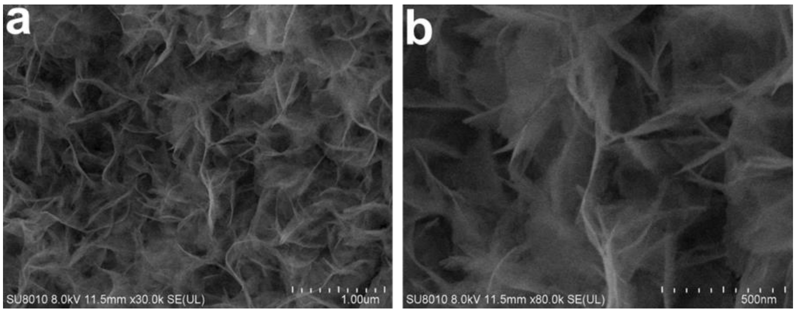

Figure S4 SEM images of MFO NS/IF.

SEM images of CFO NP/IF are shown in the **Figure S5**. CFO NP/IF exhibits morphology of nanoparticle with particle size from about 30 to 150 nm.

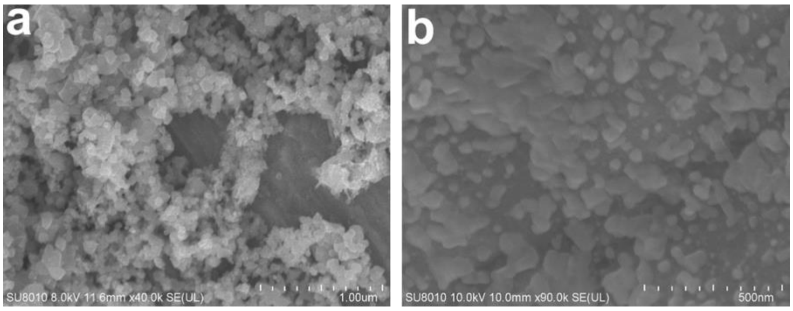

Figure S5 SEM images of CFO NP/IF.

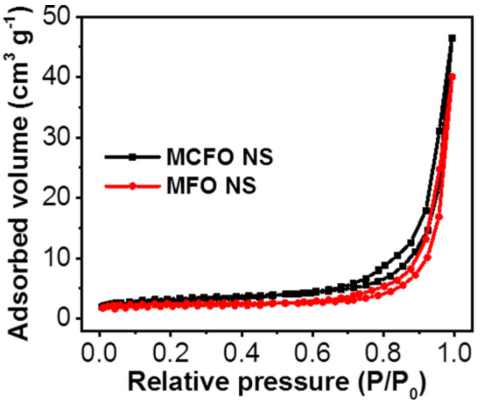

Figure S6 N_2 adsorption-desorption isotherms of MCFO NS and MFO NS.

The morphology of nanosheets has been formed as low as 70 ^{o}C at hydrothermal process (**Figure S7**). Nanosheets grow gradually with the increase of temperature and 3D flower like nanosheets form at about 90 ^{o}C. A large number of NPs are formed below 70 ^{o}C. However, the NPs gradually decompose and disappeared with temperature raised. It is due to the formation of MCFO solid solution with the decomposition of larger CFO NPs and redistribution of Co and Fe cations.

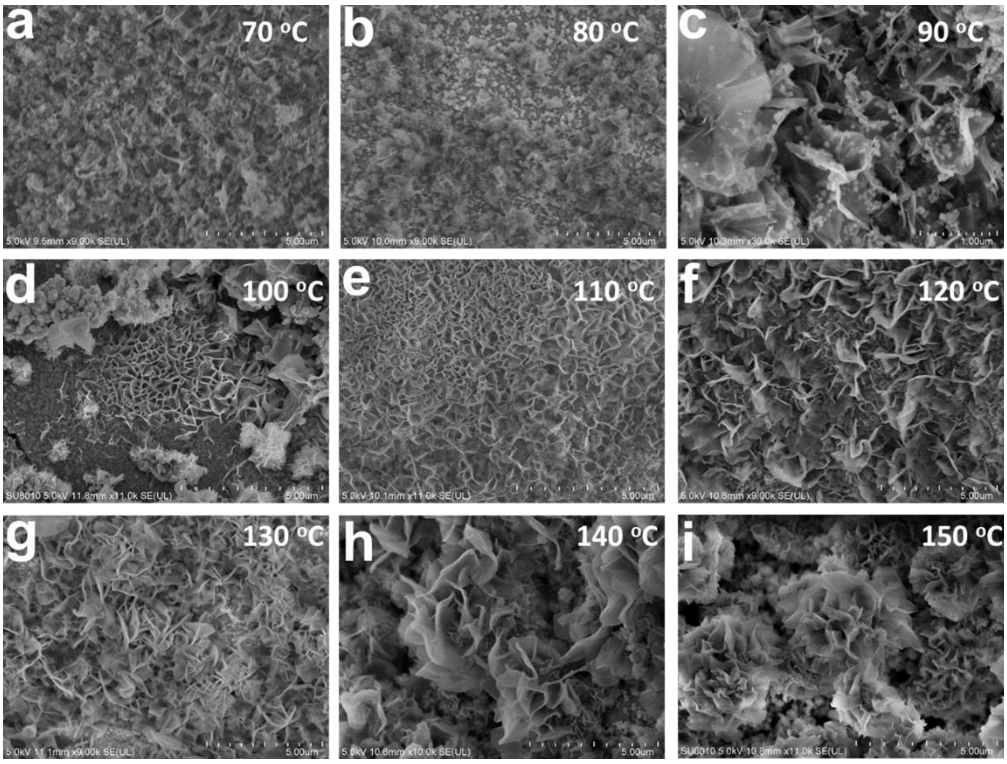

Figure S7 SEM images of samples only after hydrothermal treatment from 70 to 150 ^{o}C for 6 h, respectively.

SEM images of samples only by hydrothermal treatment are shown in the **Figure S8**. SEM

images indicate that the 3D flower like morphology of NSs has been synthesized after hydrothermal treatment. Meanwhile, NPs like morphology cannot be observed.

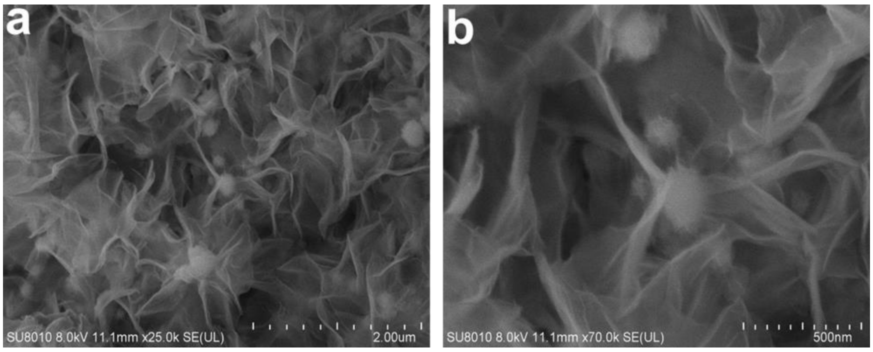

Figure S8 SEM images of samples after hydrothermal treatment at 150 ^{o}C for 6 h.

The morphologies of samples after hydrothermal treatment at 150 ^{o}C for 0-6 h were investigated by SEM characterization (**Figure S9**). The morphology of NS forms at 0 h and grows gradually with the increase of time. With the growth of NS, the three-dimensional (3D) flower-like nanosheet morphology generates at 3 h. A large number of NPs appear at 0 h and gradually grow up. After 3 h, they gradually decompose and disappear.

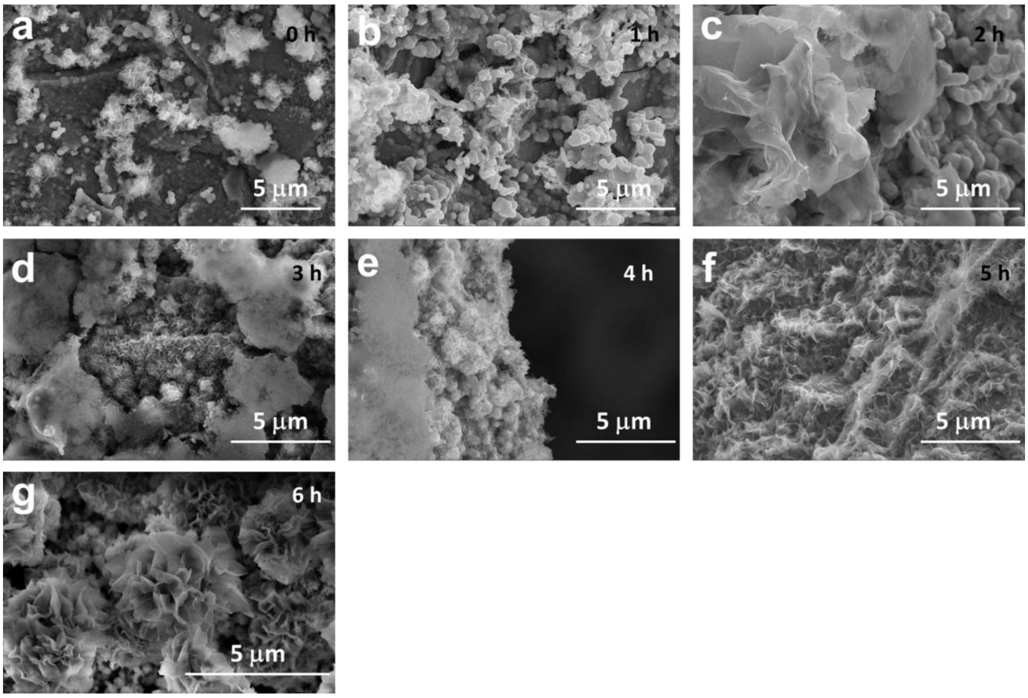

Figure S9 SEM images of samples after hydrothermal treatment at 150 ^{o}C for 0-6 h, respectively.

TEM and the electron diffraction images of MFO NS are shown in the **Figure S10**. MFO NS/IF exhibits nanosheets like morphology (**Figure S10a**). A typical high resolution (HR) TEM image of edge on MFO NS is shown in **Figure S10b**. The crystalline indices of 0.14,

0.19 and 0.21 nm are corresponding to the (442), (331) and (440) facets of MFO, respectively (**Figure S10c**). Moreover, MFO NS is composed of MFO NPs with about 5 nm.

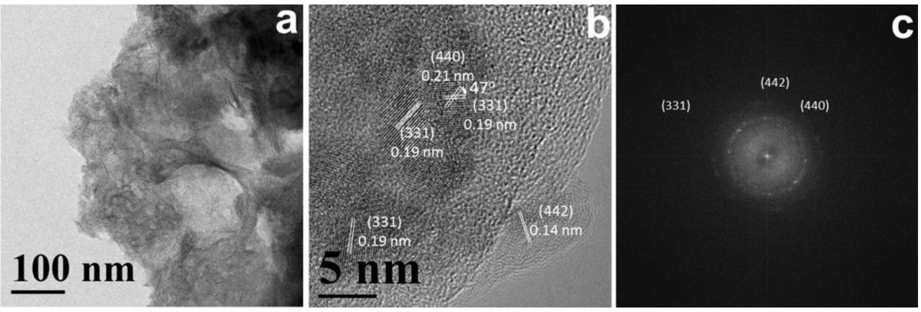

Figure S10 TEM and electron diffraction images of MFO NS/IF.

TEM and electron diffraction images of CFO NP are shown in the **Figure S11**. As results of SEM (**Figure S5**), CFO NP exhibits particle size from about 30 to a few hundred nm. HRTEM image of a typical CFO NP is shown in **Figure S11b**. Crystalline index of 0.17 nm is corresponding to (422) facet of CFO. Crystalline indices of 0.30 and 0.15 nm are corresponding to {220} and {440} facets of CFO (**Figure S11c**).

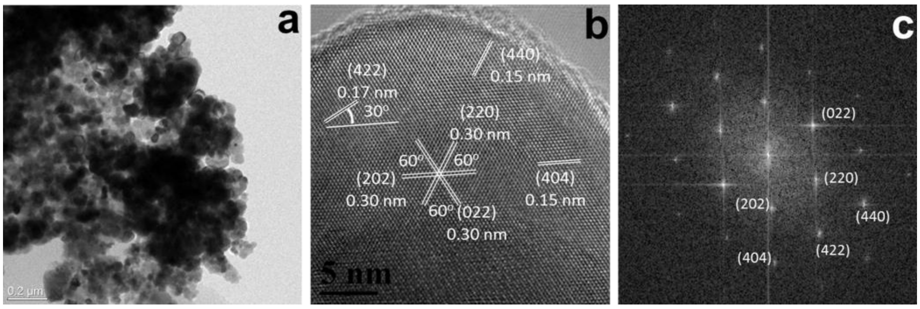

Figure S11 TEM and electron diffraction images of CFO NP/IF.

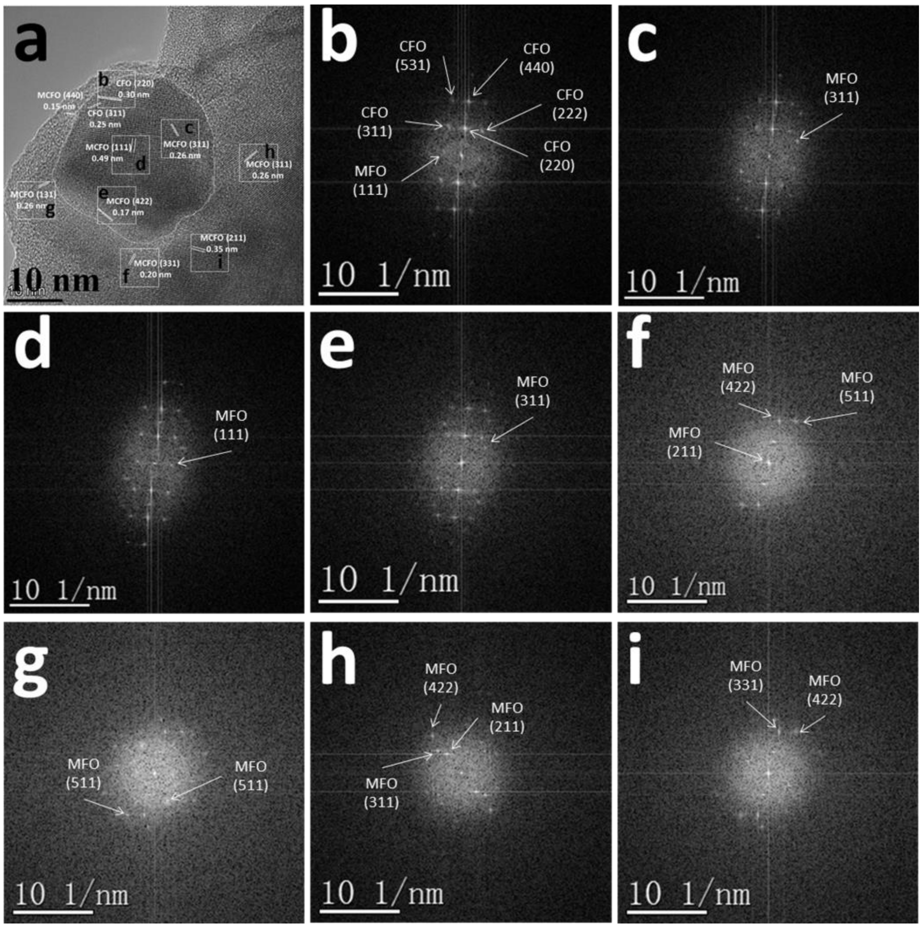

Figure S12 (a) HR-TEM image of MCFO NS/IF. (b-i) Selected-area electron diffraction images of corresponding area in **Figure S10a**.

Configurations and electronic structures of MCFO NS, MFO NS and CFO NP were characterized using X-ray photoelectron spectroscopy (XPS) (**Figure S13-16**). MCFO NS, MFO NS and CFO NP were separated from IF by ultrasonic treating in alcohol for over 6 h before XPS measurement. The XPS spectra of MCFO NS, MFO NS and CFO NP in Fe 2p are shown in **Figure S13**. In general, Fe^{2+} occupies octahedral sites and Fe^{3+} occupies both octahedral and tetrahedral sites. Binding energies (BEs) 709.4 and 722.6 eV in Fe $2p_{3/2}$ and $2p_{1/2}$ are ascribed to Fe^{2+}_{oct}; BE at 710.7 and 724.3 eV in Fe $2p_{3/2}$ and $2p_{1/2}$ are contributed to Fe^{3+}_{oct}; BEs loaded at 711.3 and 725.1 eV in Fe $2p_{3/2}$ and $2p_{1/2}$ are corresponding to Fe^{3+}_{td}.[S1] In addition, the satellite peak is loaded at 716 eV.[S2] As a result, Fe cations in MCFO NS, MFO NS and CFO NP both occupy octahedral and tetrahedral sites and mainly fill into octahedral interstices. With the formation of MCFO NS, the XPS spectrum in Fe 2p shifts to lowest BE than that of MFO NS and CFO NP. It indicates that the highest occupation of Fe_{oct} in MCFO.

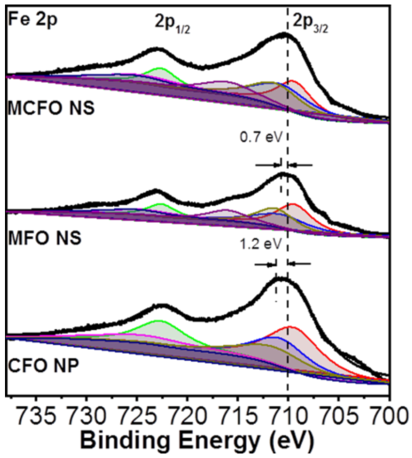

Figure S13 XPS spectra of MCFO NS, MFO NS and CFO NP in Fe 2p.

XPS spectra of MCFO NS and CFO NP in Co 2p are shown in **Figure S14**. It is considered that Co^{2+} is occupied in the tetrahedral sites and Co^{3+} is filling into octahedral sites. BEs at 781.4 and 796.8 eV in Co $2p_{3/2}$ and $2p_{1/2}$ are ascribed to Co^{2+}_{td} and BEs at 779.9 and 794.8 eV are contributed to Co^{3+}_{oct}.[S3] Two satellite peaks are loaded at 787 and 802 eV.[S4] As a result, almost all Co cations occupy the tetrahedral sites in both MCFO NS and CFO NP.

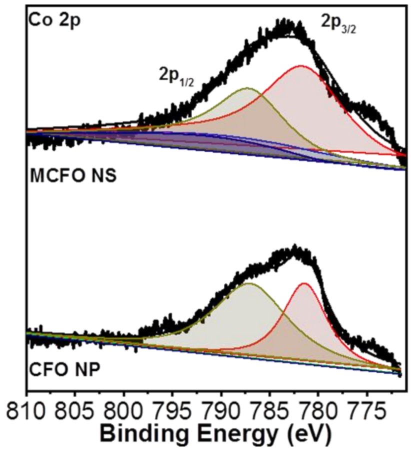

Figure S14 XPS spectra of MCFO NS and CFO NP in Co 2p.

XPS spectra of MCFO NS and MFO NS in Mo 3d can be deconvoluted into eight peaks (**Figure S15**), assigning to Mo^{2+} (228.6 and 231.8 eV), Mo^{3+} (229.4 and 232.4 eV), Mo^{4+} (231.3 and 234.5 eV) and Mo^{6+} (232.8 and 235.4 eV).[S5] The XPS spectrum of MCFO NS in Mo 3d shifted to higher BE of 0.8 eV than that of MFO NS, revealing higher valence states of Mo cations in MCFO NS. Generally, valence states of Mo cations in MFO are mainly Mo^{3+} and Mo^{4+}.[S6] It is reported that the TM_{td} is hardly detectable on the near surface.[S7] Thus, Mo^{6+} is believed mainly from the oxidation of the Mo_{oct} on the surface. It means that the occupation of Mo into octahedral sites in MCFO NS is increasing than that of MFO NS.

Meanwhile, the oxidation of Mo cations can cause the formation of Fe cation vacancies.[S8] Therefore, higher valence states of Mo cations in MCFO NS are revealing more cation vacancies. However, according to the result of Raman spectroscopy, it is considered that the dominant occupancy of Mo cation in MCFO and MFO are both in the tetrahedral site. Therefore, the MFO and MCFO are deemed as the complex spinel structures with dominant occupancy of Mo cations in the tetrahedral sites and a little amount of Mo cations filling into octahedral interstices [S9].

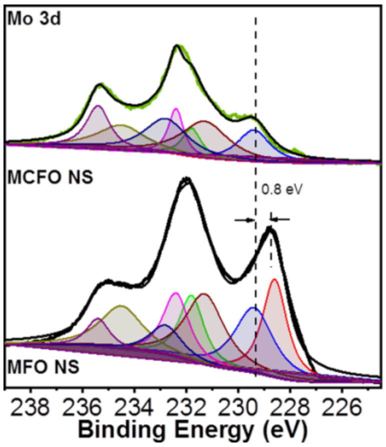

Figure S15 XPS spectra of MCFO NS and MFO NS in Mo 3d.

XPS spectra of MCFO NS, MFO NS and CFO NP in O 1s are shown in **Figure S16**. BEs in 529.9 and 530.8 eV can be assigned to lattice oxide oxygen (TM-O bonds) and surface absorbed containing species.[S10]

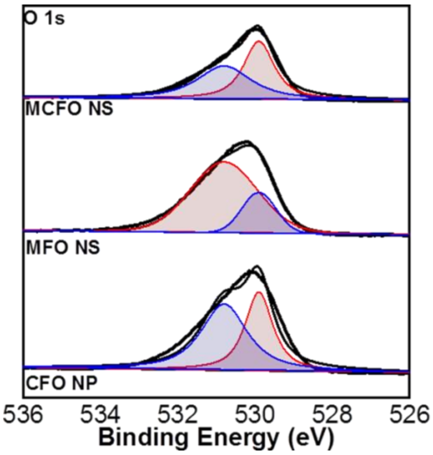

Figure S16 XPS spectra of MCFO NS, MFO NS and CFO NP in O 1s.

In the structure of cubic spinel oxide, nearest TM_{oct} cations share two O anions as edge and an O anion is shared as vertex by TM_{oct} and neighbored TM_{td}. As shown in **Figure S17**, an

unfilled octahedral interstice shares two O anions with the nearest TM_{oct} cation. However, three O anions are shared between unoccupied octahedral sites and closed TM_{td} as the face. As a result, the distance between unfilled octahedral interstice and neighbored TM_{oct} or two closed unoccupied octahedral sites is equal with TM_{oct}-TM_{oct}. While the distance between unfilled octahedral sites and TM_{td} is shorter than TM_{oct}-TM_{t}..

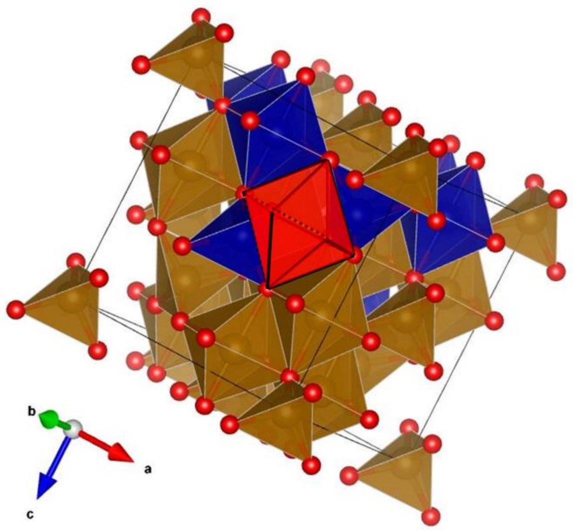

Figure S17 The crystal structure of MFO or CFO. An unfilled octahedral interstice is marked in the figure.

Figure S18 shows the polarization curves of samples heat-treated at 300, 400 and 500 ^{o}C. As a result, MCFO formed at 400 ^{o}C exhibit the best catalytic activity. It may be because of the suitable temperature for the formation of cations vacancies and refilling of Co and Fe cations to construct more active TM_{oct} sites.

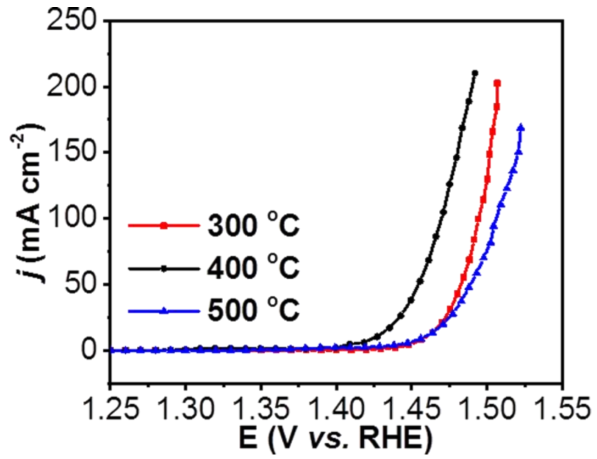

Figure S18 The polarization curves of OER on samples heat-treated at 300, 400 and 500 ^{o}C, respectively.

Polarization curves of MFO NS/IF and CFO NP/IF electrocatalysts for OER have been simply added in order to clarify the promotion of geometric engineering on MCFO NS/IF

electrocatalysts (**Figure S19**). MCFO NS/IF electrocatalysts require overpotential of only 240 mV to achieve 100 mA cm^{-2}, while polarization curve of MFO NS/IF + CFO NP/IF achieves 100 mA cm^{-2} at 1.51 V *vs*. RHE. MCFO NS/IF electrocatalysts exhibit a current density of 261 mA cm^{-2} at 1.50 V *vs*. RHE, which is about 4.2 times of the current density on MFO NS/IF + CFO NP/IF (62 mA cm^{-2}). The catalytic activity of OER on MCFO NS/IF is much better than the simple addition of activities on MFO NS/IF and CFO NP/IF, revealing the improvement of geometric engineering on MCFO NS by constructing more active sites.

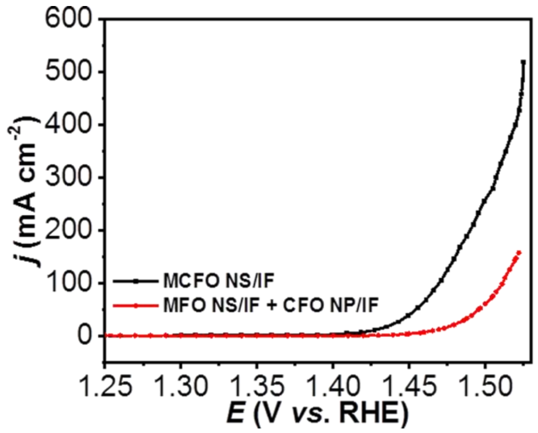

Figure S19 The polarization curves of OER on MCFO NS/IF electrocatalysts and the summation of polarization curves for OER on both MFO NS/IF and CFO NP/IF electrocatalysts (MFO NS/IF + CFO NP/IF).

It is well known that specific surface areas of electrocatalysts played an important role in the electrochemical reaction process. Thus, electrochemical surface area (ECSA) is considered an important parameter for evaluating performances of electrocatalysts and clarifying the origin of catalytic activity. Generally, ECSA is estimated through electrochemical double-layer capacitance (C_{dl}) of electrocatalysts from CV curves in a non-Faradaic region with different scan rates [S11, S12]:

$$C_{dl} = \frac{d(\Delta j)}{2dv}$$

In this formula, Δj represents the capacitive current density in selected potential on CV curves and v is corresponding to scan rate. In a word, the slope of Δj against different scan rates can be used as parameter for evaluating its ECSA [S11, S13]. Relationships between Δj against different scan rates are obtained from corresponding CV cures on various electrocatalysts in 1.0 M KOH (**Figure S20a-d**). As shown in **Figure S21**, MCFO NS/IF shows a largest slope representing to largest ECSA than comparisons. The larger ECSA of MCFO NS/IF comes

from the morphology of nanosheets, which is beneficial for exposing more active sites and leading to better catalytic performance.

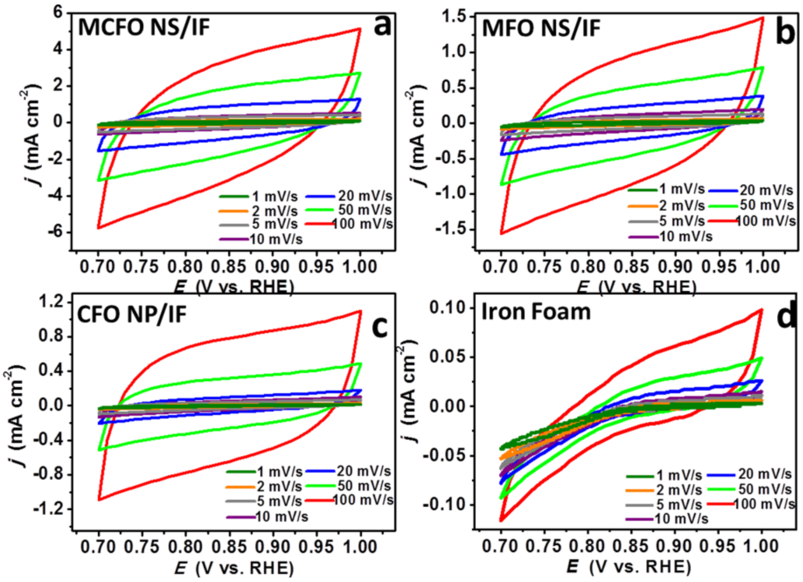

Figure S20CV curves of (a) MCFO NS/IF, (b) MFO NS/IF, (c) CFO NP/IF and (d) IF electrocatalysts in 1.0 M KOH from 0.7 to 1.0 V *vs*. RHE with scan rates from 1 to 100 mV s^{-1}.

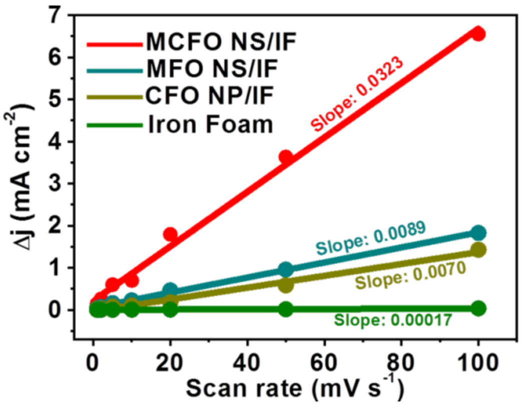

Figure S21 Relationships between capacitive current densities (*Δj*) measured on various electrocatalysts against different scan rates in 1.0 M KOH.

Nyquist plots of MCFO NS/IF, MFO NS/IF and CFO NP/IF electrocatalysts were performed to study kinetics of OER process. Experimental data determined from 1.4 – 1.6 V *vs*. RHE on various electrocatalysts in 1.0 M KOH were fitted by a two-time constant model which is always described the mechanism of OER on porous electrodes (**Figure S22 and 23**). In general, charge transfer resistance (R_{ct}) in two-time constant model is used as an important parameter for activity of electrocatalysts and found to be overpotential-dependent[S14]. MCFO NS/IF electrocatalysts exhibit lowest R_{ct} than that of MFO NS/IF and CFO NP/IF, which indicates minimum reaction resistance and faster charge transferring.

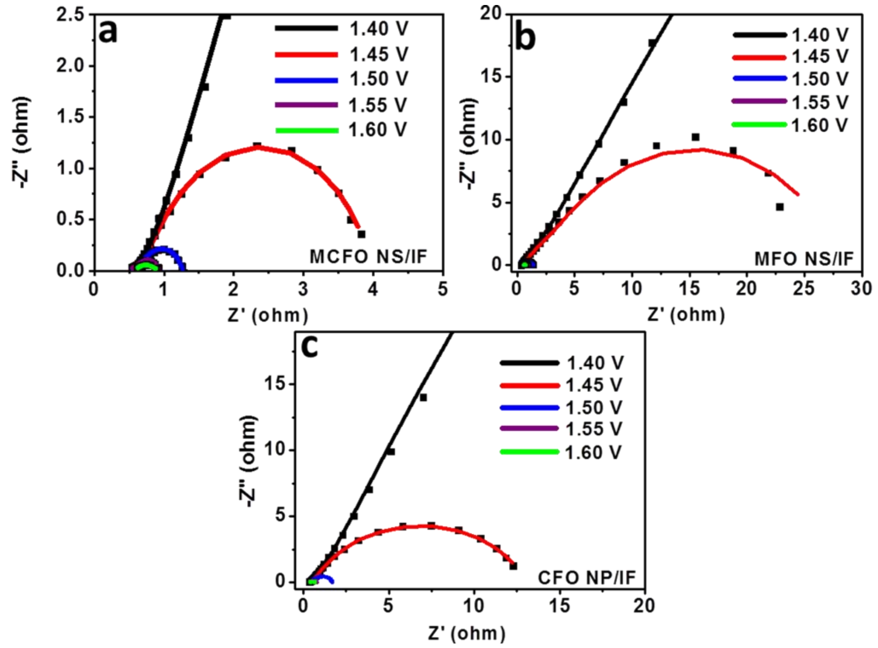

Figure S22 Nyquist plots of (a) MCFO NS/IF, (b) MFO NS/IF and (c) CFO NP/IF electrocatalysts for OER at various overpotentials in 1.0 M KOH..

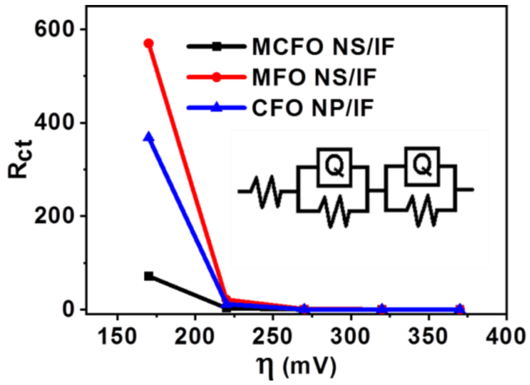

Figure S23 Relationships between resulting R_{ct} with corresponding overpotentials on various electrocatalysts in 1.0 M KOH

The XRD, XPS and SEM characterizations have been performed to investigate the structural stability of MCFO NS/IF. There is almost no change in XRD pattern, XPS spectrum and morphology of MCFO NS/IF before and after stability test, indicating a robust structural stability of MCFO NS/IF in OER process

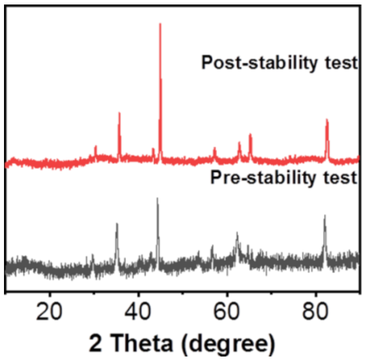

Figure S24 XRD patterns of MCFO NS/IF before and after stability test.

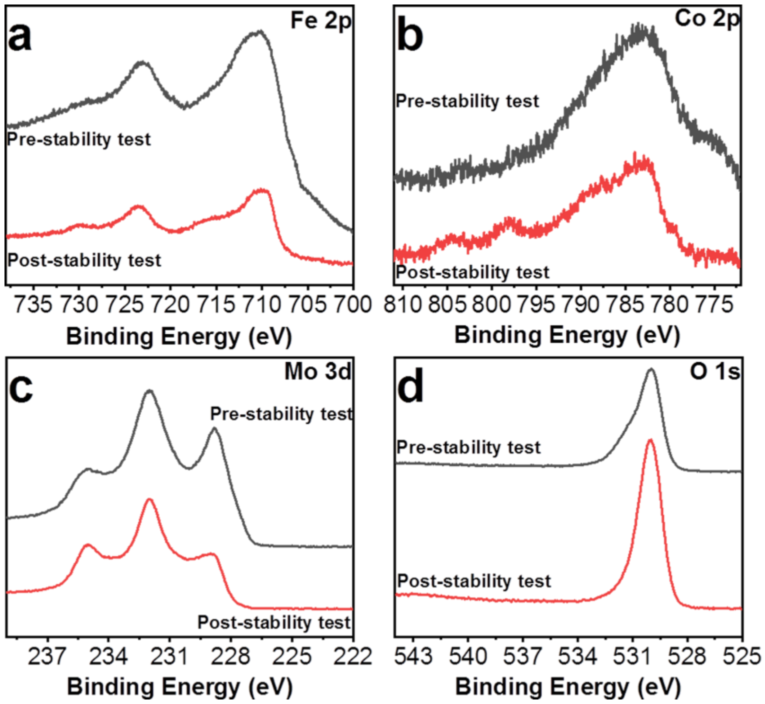

Figure S25 XPS spectra of MCFO NS/IF before and after stability test.

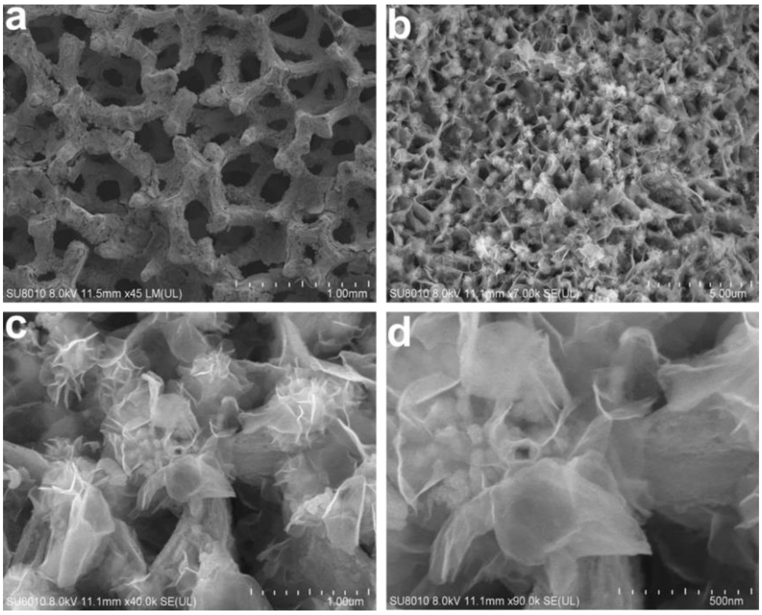

Figure S26 SEM images of MCFO NS/IF after stability test.

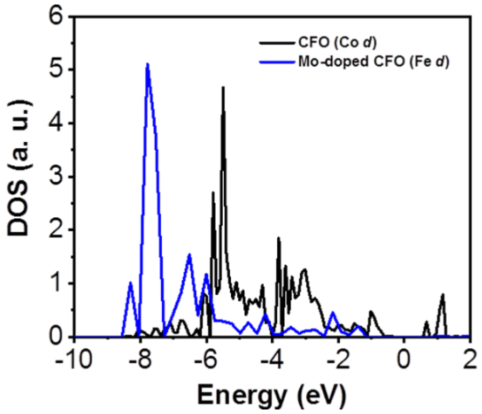

Figure S27 The electronic structure of CFO and Mo-doped CFO before reaction.

The electronic structures of Co-doped MFO and MFO were also calculated. The comparison between the overlapping of *d* orbital of metal active site and *p* orbital of O in Co-doped MFO and MFO indicates MFO shows the much stronger binding to *OH, which agrees well with the plotted Gibbs free energy diagram (**Figure S28**).

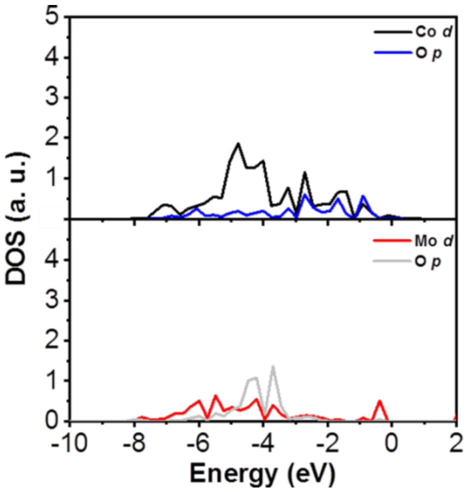

Figure S28 The curve of projected density of state (PDOS) of Co *d* orbital and O *p* orbital for *OH on Co-doped MFO (top); the curve of projected density of state (PDOS) of Mo *d* orbital and O *p* orbital for *OH on MFO.

According to the results of DFT calculations, we expect to verify whether Mo-doped CFO has a better activity for OER experimentally. Therefore, solid solutions with different ratios of Co and Mo cations were fabricated. Because the atomic ratio of Mo to Co in MCFO NS is close to 11:1, in the case of remaining the total amounts of foreign cations (Mo and Co), the samples with atomic ratios of Mo to Co of 8.5:3.5, 6:6, 3.5:8.5 and 1:11 were synthesized, respectively, and are denoted as $M_{8.5}C_{3.5}FO$ NP/IF, M_6C_6FO NP/IF, $M_{3.5}C_{8.5}FO$ NP/IF and $MC_{11}FO$ NP/IF.. **Figure S29** shows the HAADF-STEM images of these samples. As a result, $M_{8.5}C_{3.5}FO$ exhibits morphology of NPs with the size of 20-50 nm. With the increase of amounts of Co cations, the particle sizes become larger and larger. The average particle size of $MC_{11}FO$ is even more than 200 nm. The XRD patterns indicate that $M_{8.5}C_{3.5}FO$ NP/IF, M_6C_6FO NP/IF, $M_{3.5}C_{8.5}FO$ NP/IF and $MC_{11}FO$ NP/IF present the structures of spinel oxides supported on IF (**Figure S30a**). However, the performances of OER on these catalysts are both worse than that of MCFO NS/IF and only $M_{8.5}C_{3.5}FO$ NP/IF exhibits a better activity than MFO NS/IF (**Figure S30b**). In addition, with the increase of contents of Co cations, the performance of OER becomes worse and worse. The decrease of activities may be due to the loss of the electrochemically active area (ECSA). Unfortunately, it does not prove that Mo doped CFO has better activity experimentally.

Then, to study the promotion of heterostructures between MCFO NS and CFO NP on OER, the amounts of $CoCl_2{\cdot}6H_2O$ were reduced to 0.004 and 0.002 mmol when maintaining other synthetic conditions and these two samples are denoted as $M_{11}C_{0.66}FO$ NS/IF and $M_{11}C_{0.33}FO$ NS/IF. According to the HAADF-STEM images, $M_{11}C_{0.66}FO$ NS and $M_{11}C_{0.33}FO$ NS exhibit

nanosheet-like morphologies composed of smaller NPs and CFO with larger particle size can not be observed (**Figure S31**). Meanwhile, XRD patterns of $M_{11}C_{0.66}FO$ NS/IF and $M_{11}C_{0.33}FO$ NS/IF present the structures of spinel oxides supported on IF (**Figure S32a**). It indicates that the residual CFO NPs in MCFO NS are caused by excessive Co precursor and CFO NPs can be removed by reducing the amounts of Co precursor. $M_{11}C_{0.66}FO$ NS/IF and $M_{11}C_{0.33}FO$ NS/IF can achieve the current densities of 100 mA cm^{-2} for OER at 1.48 and 1.49 V *vs*. RHE, respectively, which are only 10 and 20 mV higher than that of MCFO NS/IF (*Figure S32b*). It indicates the promotion of interface for heterostructures between MCFO and CFO and appropriate component of solid solutions on activity of OER. However, the improvement is very limited, probobly due to the little amount of residual CFO NPs in MCFO NS. Meanwhile, the better catalytic activities for OER on $M_{11}C_{0.66}FO$ NS/IF and $M_{11}C_{0.33}FO$ NS/IF than MFO NS/IF imply that the promotion of performance mainly comes from the atomic-scale geometrical modification in the solid solutions of spinel oxides. Thus, in this work, the model of Mo-doped CFO has been constructed to describe the interface of heterostructures between MCFO and CFO. The model of Co-doped MFO is used to describe not only the solid solution of MCFO, but also the interface of heterostructures between MCFO and CFO.

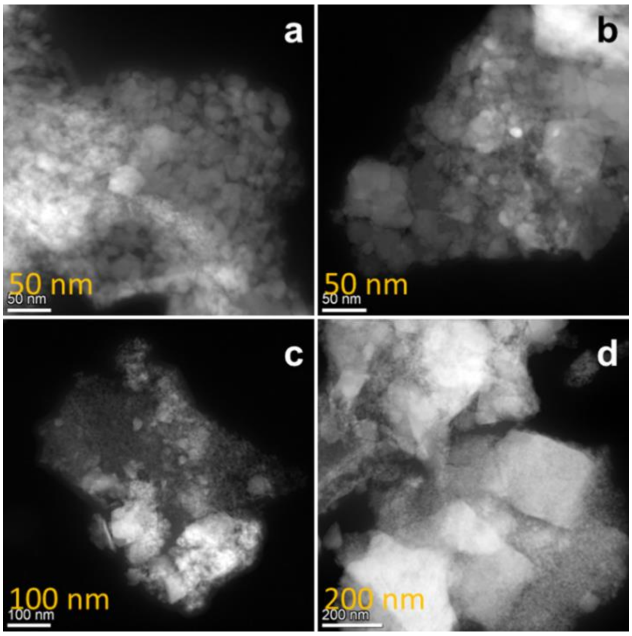

Figure S29 HAADF-STEM images of (a) $M_{8.5}C_{3.5}FO$ NP, (b) M_6C_6FO NP, (c) $M_{3.5}C_{8.5}FO$ NP and (d) $MC_{11}FO$ NP.

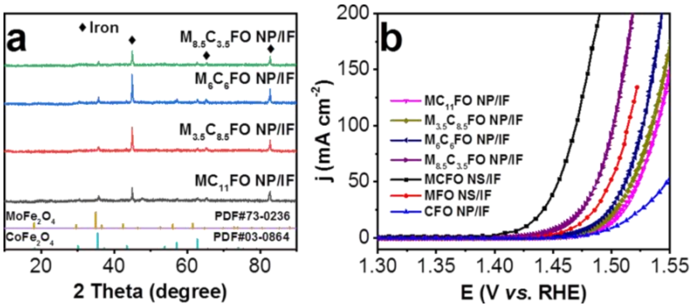

Figure S30 (a) XRD patterns of $M_{8.5}C_{3.5}FO$ NP/IF, M_6C_6FO NP/IF, $M_{3.5}C_{8.5}FO$ NP/IF and $MC_{11}FO$ NP/IF. (b) LSV curves of OER on MCFO NS/IF, MFO NS/IF, CFO NP/IF, $M_{8.5}C_{3.5}FO$ NP/IF, M_6C_6FO NP/IF, $M_{3.5}C_{8.5}FO$ NP/IF and $MC_{11}FO$ NP/IF.

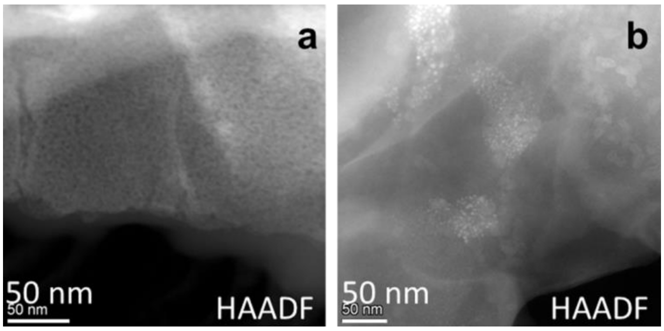

Figure S31 The high angle annular dark field-scanning transmission electron microscope (HAADF-STEM) images of (a) $M_{11}C_{0.66}FO$ NS and (b) $M_{11}C_{0.33}FO$ NS.

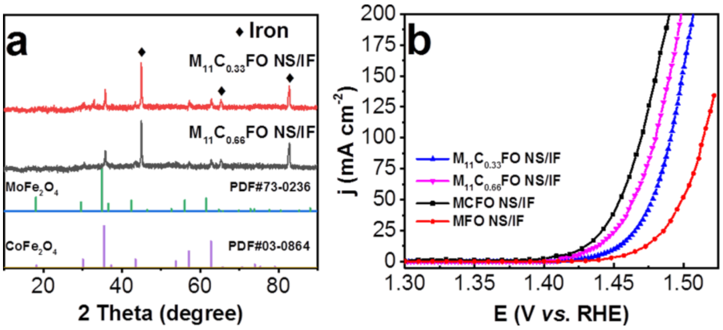

Figure S32 (a) XRD patterns of $M_{11}C_{0.66}FO$ NS/IF and $M_{11}C_{0.33}FO$ NS/IF. (b) LSV curves of OER on $M_{11}C_{0.66}FO$ NS/IF, $M_{11}C_{0.33}FO$ NS/IF, MCFO NS/IF and MFO NS/IF, respectively.

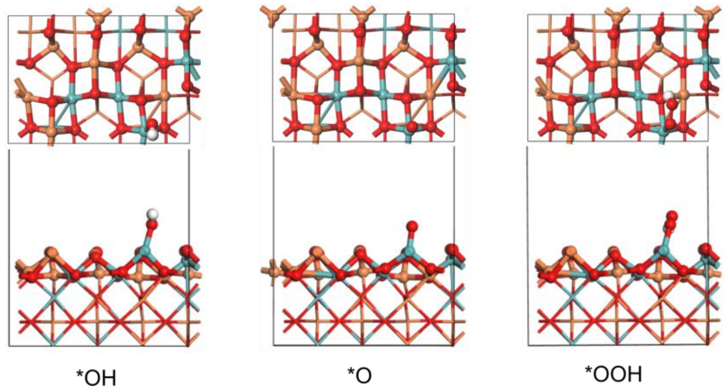

Figure S33 The optimized structures of key intermediates during OER on MFO. Color code: orange, Fe; teal, Mo; red, O; white, H.

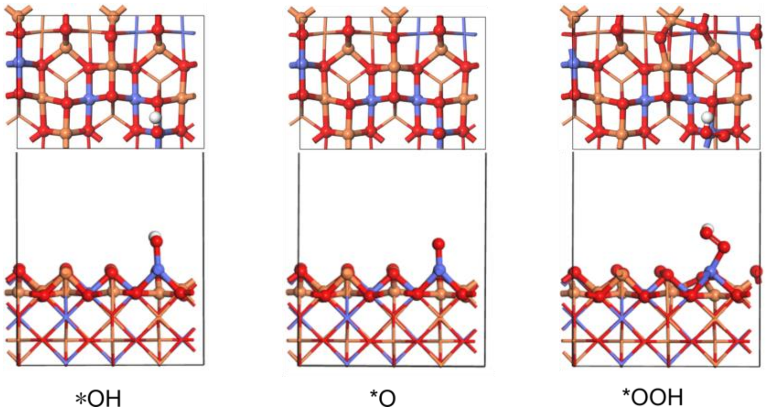

Figure S34 The optimized structures of key intermediates during OER on CFO. Color code: orange, Fe; blue, Co; red, O; white, H.

In the synthesis process, the used amounts of $(NH_4)_6Mo_7O_{24}{\cdot}4H_2O$, as the precursor of Mo cations, and $CoCl_2{\cdot}6H_2O$, as the precursor of Co cations, were 0.010 and 0.006 mmol, respectively. The atomic ratio of Mo to Co is about 11.7:1. To investigate the atomic ratios of cations in MCFO NS, MFO NS and CFO NP, the Inductively Coupled Plasma-atomic Emission Spectrometry (ICP) was characterized. Firstly, MCFO NS, MFO NS and CFO NP were separated from IF by ultrasonic treating in alcohol for over 12 h and then dried in vacuum oven for 12 h. Secondly, 5 mg of MCFO NS, MFO NS and CFO NP were leached in 100 mL aqua regia, respectively, and then the solutions were diluted and detected by ICP characterization. As shown in **Table S1**, the atomic ratio of Fe:Mo:Co in MCFO NS is about 25.80:10.94:1. Meanwhile, the atomic ratios of Fe:Mo in MFO NS is about 2.24:1 and Fe:Co in CFO NP is about 2.11: 1, respectively. The contents of Fe cations are slightly higher than the theoretical value in all samples, probably because a little amount of metallic Fe was

separated from IF during the ultrasonic treatment. As a result, the atomic ratio of Mo to Co in MCFO NS is close to 11:1. In addition, ICP was further used to explore the contents of Mo and Co cations in as-prepared samples. Firstly, MCFO NS/IF, MFO NS/IF and CFO NP/IF with sizes of 2.0×2.0 cm, were leached in 100 mL aqua regia, respectively, and then the solutions were diluted and characterized by ICP. As shown in **Table S2**, the productivities of Mo and Co cations in MCFO NS/IF are 87.55 and 93.33%, respectively. At the same time, those in MFO NS/IF and CFO NP/IF are 89.70 and 94.46%, respectively. It indicates that the contents of Mo cations in MFO NS/IF and Co cations in CFO NP/IF are very close to that in the MCFO NS/IF and they are reasonable as comparisons.

Table S1 Cationic contents and atomic ratios of various cations in MCFO NS, MFO NS and CFO NP detected by ICP characterization.

	Fe		Mo		Co	
	mg	at%	mg	at%	mg	at%
MCFO	2.159	68.36	1.573	28.99	0.089	2.65
MFO	2.304	69.14	1.767	30.86	N/A	N/A
CFO	2.521	67.85	N/A	N/A	1.260	32.15

Table S2 Contents and productivities of various cations in MCFO NS/IF, MFO NS/IF and CFO NP/IF detected by ICP characterization.

	Fe		Mo		Co	
	mg	%	mg	%	mg	%
MCFO	379.364	N/A	5.880	87.55	0.330	93.33
MFO	388.935	N/A	6.025	89.70	N/A	N/A
CFO	384.915	N/A	N/A	N/A	0.334	94.46

Table S3 Comparison of OER activities on MCFO NS/IF and recently reported state-of-the-art electrocatalysts

Catalysts	η_{10} (mV)	η_{100} (mV)	Electrolyte	Tafel Slope (mV/dec)	Ref.
**MCFO NS/IF**	180	240	1 M KOH	38	This work

**MFO NS/IF**	230	290	1 M KOH	41	This work
**CFO NP/IF**	250	N.A.	1 M KOH	59	This work
**NiMo-FG**	338	390	1 M KOH	67.0	*ACS Catal.*, **2020**, *10*, 792
**Fe-Ni(OH)$_2$**	219	273	1 M KOH	53.0	*ACS Energy Lett.*, **2019**, *4*, 622
**Fe$_{MOFs}$-SO$_3$**	218	255	1 M KOH	36.2	*Adv. Energy Mater.*, **2020**, *10*, 2000184
**NiO/CN**	261	350	1 M KOH	58.9	*Adv. Funct. Mater.*, **2019**, *29*, 1904020
**(NiCo)Fe-MOF-NF**	257	290	1 M KOH	41.3	*Adv. Mater.*, **2019**, *31*, 1901139
**CoO/Co$_3$O$_4$**	270	315	1 M KOH	55.0	*Angew. Chem.*, **2020**, *132*, 6996
**hcp-NiFe@NC**	226	263	1 M KOH	41.0	*Angew. Chem. Int. Ed.*, **2019**, *58*, 6099
**Co$_{1.2}$Fe/C**	260	320	1 M KOH	45.2	*Adv. Sci.*, **2019**, *6*, 1900117
**Fe@BIF-91**	350	380	1 M KOH	71	*Adv. Sci.*, **2019**, *6*, 1801920
**NiFe$_{0.5}$Sn-A**	270	300	1 M KOH	50	*Adv. Sci.*, **2020**, *7*, 1903777
**P/Mo-Co$_3$O$_4$@CC**	265	330	1 M KOH	59.4	*Adv. Sci.*, **2020**, *7*, 1902830
**CoFeN NSs**	266	300	1 M KOH	30.0	*Nano Energy*, **2018**, *57*, 644
**FeNiP/C**	229	310	1 M KOH	74.5	*Nano Energy*, **2019**, *62*, 745
**CoFeP$_x$**	323	390	1 M KOH	58.0	*Nano Energy*, **2019**, *63*, 103855
**NiFe-LDH/MXene/NF**	229	275	1 M KOH	44	*Nano Energy*, **2019**, *63*, 103880
**Co BDC-Fc-NF**	178	241	1 M KOH	51	*Nat. Commun.*, **2019**, *10*, 5048
**Ru/CoFe-LDHs**	198	245	1 M KOH	39	*Nat. Commun.*, **2019**, *10*, 1711
**Ni-O-G SACs**	224	380	1 M KOH	42	*Adv. Sci.*, **2020**, *7*, 1903089
**MIL-53(FeNi)/NF**	205	244	1 M KOH	31.4	*Adv. Energy Mater.*, **2018**, *8*, 1800584
**Co$_3$O$_4$/Co-Fe**	297	360	1 M KOH	61	*Adv. Mater.*, **2018**, *30*, 1801211

Table S4 The *d*-band center of Fe *d* orbital for *OH on Mo-doped CFO and Co *d* orbital for *OH on CFO.

	Mo-doped CFO	CFO
d-band center (eV)	-6.11	-4.17

The active site was determined according to the screening results of the binding energy of *OH on various possible adsorption sites on both Mo-doped CFO and CFO. The most possible active site should be the metal site with the largest binding energy to *OH, which is the very first intermediate during OER. The screening results are summarized into **Table S5**.

Table S5 The summarized data of *OH binding energy (ΔE_b/eV) on Mo-doped CFO and CFO. *The metal sites are determined by considering the structure symmetry and metal site coordination.

*OH	Mo-doped CFO				CFO			
Metal sites*	**Fe1**	Fe2	Co	Mo	Fe1	Fe2	**Co1**	Co2
E_{total}	-464.30	-462.22	-461.59	-462.49	-433.25	-434.05	-435.53	-432.69
E_{slab}	-453.92	-453.92	-453.92	-453.92	-423.60	-423.60	-423.60	-423.60
ΔE_b	**-3.22**	-1.14	-0.51	-1.42	-2.49	-3.29	**-4.77**	-1.93

Reference

[S1] a) M. Wen, Q. Li, Y. Li, *J. Electron. Spectrosc. Relat. Phenom.* **2006**, *153*, 65; b) J. Geng, J. Liu, J. Yan, M. Ba, Z. He, Y. Li, *Int. J. Corrosion* **2018**, *2018*, 7479383 .

[S2] P. Mills, J. Sullivan, *J. Phys. D: Appl. Phys.* **1983**, *16*, 723.

[S3] a) J. Shi, H. Fan, X. Liu, A. J. Bell, *J. Am. Ceram. Soc.* **2014**, *97*, 848; b) T. W. Kim, M. A. Woo, M. Regis, K.-S. Choi, *J. Phys. Chem. Lett.* **2014**, *5*, 2370; c) P. Wang, H. Zhou, C. Meng, Z. Wang, K. Akhtar, A. Yuan, *Chem. Eng. J.* **2019**, *369*, 57.

[S4] Y. Zhong, Y. Wu, B. Chang, Z. Ai, K. Zhang, Y. Shao, L. Zhang, X. Hao, *J. Mater. Chem. A* **2019**, *7*, 14638.

[S5] a) Y. Chen, Y. Zhang, Y. Ma, T. Tang, Z. Dai, J. Hu, L. Wan, *Chin. J. Chem.* **2017**, *35*, 911; b) S. Sunu, E. Prabhu, V. Jayaraman, K. Gnanasekar, T. Seshagiri, T. Gnanasekaran, *Sensors Actuators B: Chem.* **2004**, *101*, 161.

[S6] a) A. Roy, J. Ghose, *J. Solid State Chem.* **1998**, *140*, 56; b) M. Abe, M. Kawachi, S. Nomura, *J. Phys. Soc. Jpn.* **1972**, *33*, 1296; c) T. Katayama, Y. Kurauchi, S. Mo, K. Gu, A. Chikamatsu, L. Galiullina, T. Hasegawa, *Cryst. Growth Des.* **2018**, *19*, 902; d) B. Domenichini, B. Gillot, P. Tailhades, *Thermochim. Acta* **1992**, *205*, 259; e) M. Gupta, A. Sinha, S. Kanetkar, S. Date, A. Nigavekar, *J. Phys. C: Solid State Phys.* **1979**, *12*, 2401.

[S7] a) M. Anantharaman, S. Reijne, J. Jacobs, H. Brongersma, R. Smits, K. Seshan, *J. Mater. Sci.* **1999**, *34*, 4279; b) M. Shelef, M. Wheeler, H. Yao, *Surf. Sci.* **1975**, *47*, 697.

[S8] a) B. P. Hahn, J. W. Long, A. N. Mansour, K. A. Pettigrew, M. S. Osofsky, D. R. Rolison, *Energy Environ. Sci.* **2011**, *4*, 1495; b) P. Gao, Z. Chen, Y. Gong, R. Zhang, H. Liu, P. Tang, X. Chen, S. Passerini, J. Liu, *Adv. Energy Mater.* **2020**, *10*, 1903780; c) P. Tailhades, B. Gillot, A. Rousset, *J. Phys. IV* **1997**, *7*, C1; d) L. Bouet, P. Tailhades, A. Rousset, B. Domenichini, B. Gillot, *Solid State Ionics* **1992**, *52*, 285.

[S9] Q. Zhao, Z. Yan, C. Chen, J. Chen, *Chem. Rev.* **2017**, *117*, 10121.

[S10] W. Q. Fang, B. Zhang, H. G. Yang, *J. Alloys Compd.* **2013**, *550*, 348.

[S11] H.-P. Guo, B.-Y. Ruan, W.-B. Luo, J. Deng, J.-Z. Wang, H.-K. Liu, S.-X. Dou, *ACS Catal.* **2018**, *8*, 9686.

[S12] X. Yue, S. Huang, Y. Jin, P. K. Shen, *Catal. Sci. Technol.* **2017**, *7*, 2228.

[S13] J. Sanetuntikul, S. Hyun, P. Ganesan, S. Shanmugam, *J. Mater. Chem. A* **2018**, *6*, 24078.

[S14] a) X. Yue, S. Huang, J. Cai, Y. Jin, P. K. Shen, *J. Mater. Chem. A* **2017**, *5*, 7784; b) Y. Jin, H. Wang, J. Li, X. Yue, Y. Han, P. K. Shen, Y. Cui, *Adv. Mater.* **2016**, *28*, 3785.
